# Reliable cognitive changes the first year following guideline-based treatment of isocitrate dehydrogenase mutated gliomas: A longitudinal multicenter study

**DOI:** 10.1093/neuonc/noaf263

**Published:** 2025-11-09

**Authors:** Isabelle Rydén, Francesco Latini, Åsa Alberius Munkhammar, Per Hellström, Alice Neimantaite, Andrew Lycett, Dima Harba, Louise Carstam, Malin Blomstrand, Maria Zetterling, Anja Smits, Asgeir S Jakola

**Affiliations:** Department of Clinical Neuroscience, Institute of Neuroscience and Physiology, Sahlgrenska Academy, University of Gothenburg, Gothenburg (I.R., P.H., A.N., A.L., D.H., L.C., A.S., A.S.J.); Department of Neurology, Sahlgrenska University Hospital, Gothenburg (I.R., A.S.); Section of Neurosurgery, Department of Neuroscience, Uppsala University, Uppsala (F.L.); Rehabilitation and Pain Centre, Uppsala University Hospital, Uppsala (Å.A.M.); Department of Clinical Neuroscience, Institute of Neuroscience and Physiology, Sahlgrenska Academy, University of Gothenburg, Gothenburg (I.R., P.H., A.N., A.L., D.H., L.C., A.S., A.S.J.); Department of Clinical Neuroscience, Institute of Neuroscience and Physiology, Sahlgrenska Academy, University of Gothenburg, Gothenburg (I.R., P.H., A.N., A.L., D.H., L.C., A.S., A.S.J.); Department of Clinical Neuroscience, Institute of Neuroscience and Physiology, Sahlgrenska Academy, University of Gothenburg, Gothenburg (I.R., P.H., A.N., A.L., D.H., L.C., A.S., A.S.J.); Department of Clinical Neuroscience, Institute of Neuroscience and Physiology, Sahlgrenska Academy, University of Gothenburg, Gothenburg (I.R., P.H., A.N., A.L., D.H., L.C., A.S., A.S.J.); Department of Clinical Neuroscience, Institute of Neuroscience and Physiology, Sahlgrenska Academy, University of Gothenburg, Gothenburg (I.R., P.H., A.N., A.L., D.H., L.C., A.S., A.S.J.); Department of Neurosurgery, Sahlgrenska University Hospital, Gothenburg (L.C., A.S.J.); Department of Oncology, Institute of Clinical Sciences, Sahlgrenska Academy, University of Gothenburg, Gothenburg (M.B.); Section of Neurosurgery, Department of Neuroscience, Uppsala University, Uppsala (F.L.); Department of Clinical Neuroscience, Institute of Neuroscience and Physiology, Sahlgrenska Academy, University of Gothenburg, Gothenburg (I.R., P.H., A.N., A.L., D.H., L.C., A.S., A.S.J.); Department of Neurology, Sahlgrenska University Hospital, Gothenburg (I.R., A.S.); Department of Clinical Neuroscience, Institute of Neuroscience and Physiology, Sahlgrenska Academy, University of Gothenburg, Gothenburg (I.R., P.H., A.N., A.L., D.H., L.C., A.S., A.S.J.); Department of Neurosurgery, Sahlgrenska University Hospital, Gothenburg (L.C., A.S.J.)

**Keywords:** cognition, IDH mutation, low-grade glioma, reliable change index, neurosurgery

## Abstract

**Background:**

Management of *isocitrate dehydrogenase* (*IDH*) mutated gliomas is multidisciplinary and current guidelines support early multimodal treatment. The effects of this treatment approach on cognition are less studied. We aimed to study changes in cognitive functioning the first year following treatment in these patients and explore predictors of cognitive deterioration.

**Methods:**

Patients with a first-time diagnosis of *IDH* mutated glioma were neuropsychologically assessed before and one year after surgery. Results were compared to published norms, and impairment defined as z <−1.645. Matched controls were assessed at corresponding times, and reliable change indices (RCIs) were calculated to account for practice effects. Logistic regression models investigated predictors for cognitive declines. Tumor locations for declined versus non-declined patients were visualized using heatmaps.

**Results:**

Of the 127 included patients, 104 underwent multi-modal treatment. Pre-operative impairments ranged from 3% to 24%, depending on the specific test. Cognitive declines according to RCI domains were largest in tests of executive functioning (24%), learning/memory (23%), and language (21%). Tests of inhibition/flexibility (32%), naming speed (29%), verbal memory (28%), object naming (28%), and verbal fluency (22%) showed the largest proportions of declines. Regression models revealed that older age and chemoradiotherapy predicted declines in specific domains as well as in individual tests (*P *< 0.05).

**Conclusions:**

Significant changes occurred in several cognitive domains after guideline-based treatment. Older age and chemoradiotherapy increased the risk of cognitive declines one year after surgery, but to which extent the deficits are persistent or progressing remains unknown.

Key Points

- Patients with *IDH* mutated glioma often show cognitive impairments before treatment.
- Significant declines occur in several cognitive domains following treatment.
- Older age and early multimodal treatment are linked to cognitive declines one year postoperatively.

Importance of the Study

This study presents new insights into cognitive changes following standard multimodal treatment for *IDH* mutated glioma, prior to the widespread introduction of *IDH* inhibitors. To account for random and learning effects that occur with longitudinal testing we used reliable change indices. Individual cognitive declines were frequent across several cognitive domains one year postoperatively compared to baseline. Older age and chemoradiotherapy increased the risk of cognitive declines one year postoperatively. Identifying risk factors for cognitive decline enables treatment strategies to be tailored to patient risk profiles for cognitive deterioration.

Based on the proven survival benefit of early surgery and the administration of chemoradiotherapy, patients with *isocitrate dehydrogenase* (*IDH*) mutated gliomas now often receive early and extensive multimodal treatment, including both surgery and chemoradiotherapy, in line with the latest European Association of Neuro-Oncology guidelines.^1–5^ While the disease in this patient group can be stable for years following treatment, studies on the cognitive impact of treatment are lacking. Therefore, it remains unclear whether and how cognition is affected. In clinical practice, weighing the survival benefit of treatment versus the functional consequences can be crucial for patients and is one key parameter when determining the onco-functional balance.^6^

Patients with *IDH* mutated grade 2-3 gliomas often present with subtle or gradually worsening symptoms. Symptoms and cognitive deficits vary depending on tumor location within the brain.^7^^,^^8^ Beyond tumor location, the tumor burden, and perhaps more importantly, the tumor growth rate have been demonstrated to be associated with cognition. Faster growing tumors can outpace the brain’s capacity to compensate, while slower progression allows plasticity thereby reducing the severity of symptoms.^8^^,^^9^ Other background factors such as age, sex, education, psychological health, mental fatigue, and overall functional status are known to influence results in cognitive testing.^10^^,^^11^ Cognitive symptoms may be related to the treatment, and can occur across multiple cognitive domains. A recent meta-analysis highlighted that impairments in processing speed, attention, verbal fluency, and executive functioning following treatment were particularly common in patients with various types of glioma.^12^

Earlier studies, from the pre-molecular era, often lack information on *IDH* status, and include patients with *IDH* wild-type gliomas.^13–19^ This is problematic, as the inclusion of *IDH* wild-type glioblastomas, characterized by different preferential brain locations, earlier progression and more rapid clinical deterioration, can significantly alter the results.^20^ Longitudinal studies in unselected patients with *IDH* mutated gliomas are scarce. Instead, most studies have relied on screening instruments such as the MMSE, or been limited to post-operative assessments only.^19^^,^^21^^,^^22^ Other studies have focused on selected specific subgroups, such as patients undergoing awake surgery.^20^^,^^23^ Hence, a longitudinal study focusing solely on *IDH* mutated gliomas in an unselected cohort undergoing guideline-based treatment is needed to optimize patient counseling and clinical decision making.

In this longitudinal prospective study stretching from pre-operative to one-year post-operatively, we report on reliable cognitive changes in patients with *IDH* mutated gliomas receiving treatment according to current guidelines. Our hypothesis is that a proportion of patients experience measurable cognitive decline over the first postoperative year, and that such changes can be predicted by clinical characteristics, tumor-related factors, and treatment variables.

## Methods

### Participants

Patients were recruited between January 2016 and May 2024 via two population-based Swedish centers: Sahlgrenska University Hospital in Gothenburg and Uppsala University Hospital in Uppsala. Patients were identified via multidisciplinary team conferences at the respective site. Patients planned for either biopsy or resection were invited when presenting with a suspected diffuse lower-grade glioma defined as a hyperintense mass lesion in T2-FLAIR weighted MR images and without significant contrast enhancement. All patients were assessed in person, and included patients provided written informed consent. Data on demographics, treatment, and tumor characteristics were collected from medical records. Tumor volumes were calculated from semi-automated segmentations in T2-FLAIR weighted images preoperatively and early (<72 h) post-operatively as described previously.^24^^,^^25^ Focal neurological deficits were reported in accordance with previous studies, with deficits considered permanent if they persisted beyond three months post-operatively.^26^ Patients were neuropsychologically assessed prior to surgery, and in case of a verified *IDH* mutated glioma, also assessed one year post-operatively.

### Control Group

To provide measures of significant changes in individual test results compared to variations observed in a control group, a reliable change index (RCI) was used. A group of matched controls were assessed in person at corresponding time points as for the patients. This group comprised adults (*n* = 88) matching the patients characteristics in terms of sex, age (20-60 years), and education (8-21 years). Controls were recruited through public advertisements on social media, word-of-mouth, and posters in public places, as well as within organizations and companies. All controls provided written informed consent and filled out a health questionnaire.

### Neuropsychological Assessments

Neuropsychological assessments of patients were performed by a clinical neuropsychologist (mainly I.R. and Å.A.M). Patients were asked to fill out self-assessment forms before the visit and bring to the neuropsychologist. Self-assessments included the Hospital Anxiety and Depression Scale (HADS) that was used to evaluate symptoms of depression and anxiety. A score >8 was considered indicative of problematic symptoms of depression or anxiety.^27^ At the Uppsala site, language was assessed by a licensed speech and language pathologist. The following tests and test variables were used: Brief Visuospatial Memory Test—Revised (BVMT-R) total learning, and delayed recall, Rey Auditory Verbal Learning Test (RAVLT) total learning, and delayed recall, Rey Complex Figure (RCFT), score on copy, Boston Naming Test 60 item (BNT) total correct items, Delis-Kaplan Executive Function System (D-KEFS) Verbal Fluency: Phonemic fluency total score, and Semantic fluency (only animals) total score, Wechsler Adult Intelligence Scale (WAIS-IV) Digit span forward max items, and backward max items, WAIS-IV Coding, total score, Trail-Making Test A (TMT A) time, and B (TMT B) time, D-KEFS Color Word Interference Test (CWIT) 1-4, time for each subtest. To lower the risk for learning effects, parallel equivalent versions were used when possible (RAVLT and BVMT-R). Starting versions were randomly mixed to further avoid bias. Further descriptions of the tests can be found in Table S1.

Patients who did not have Swedish as their primary language were excluded from language dependent tests (*n* = 5). One patient with motor deficit was excluded from “pen-and-paper” tests. The test batteries were not completely uniform between the sites. The CWIT was introduced in Uppsala in 2019. Also, different versions of the digit span test were administered. To address this, only the number of correct items was used as a variable. Additionally, the coding test and the trail-making test were assessed in different versions at the two centers and were not considered equivalent. Hence, data from these tests were included only from the Gothenburg cohort.

### Statistical Analyses

Statistical analyses were performed using the Statistical Package for the Social Sciences (SPSS) version 28.0 (IBM Corporation). All tests were two-sided and a *P* value <0.05 was considered significant.

Patients’ demographic, tumor, and treatment characteristics were summarized using descriptive statistics. Raw scores from the pre-operative and one-year post-operative assessments were z-transformed according to published normative data. References for the normative data are found in Table S1. To compare our results with previous studies we calculated the proportions of patients considered to have a cognitive impairment.^23^^,^^28^ Similar to previous studies, a z-score below −1.645 (corresponding to the fifth percentile) was considered an impairment. As multiple tests increase the risk of chance deviations, we used Monte Carlo simulations to test whether the observed impairment rates were greater than expected by chance.^29^

#### Assessing change

To statistically compare proportions, Fishers exact test was used. Paired sample T-test was applied to assess within-patient differences (changes) in cases where data were normally distributed according to the Kolmogorov-Smirnov test. When data was skewed, the non-parametric Wilcoxon signed rank test was used.

To assess RCI, results from repeated assessments of our own control group were used. The regression-based RCI method presented by Maassen et al. was applied, as previously done by others.^30^^,^^31^ This specific RCI estimates whether an individual’s change in test performance exceeds what would be expected from measurement error, natural variability, or practice effects. It incorporates test-retest reliability, individual baseline scores, and variability observed in the healthy control group, adjusting for expected improvements and regression to the mean. Each patient’s results were calculated in relation to the RCIs. A significant change was defined as an RCI value exceeding 1.645 corresponding to the fifth percentile (90% CI, two-tailed α = 0.10%). Domain-level declines were calculated using the average RCI scores within each domain. The following domains were included: learning/memory, visuo-perceptual, language, executive, and speed/attention. The tests included in each domain followed the same categorization used throughout the study. Further explanation on the RCI method can be found in the Supplementary Material. This methodology has been recommended by the international cognition and ­cancer task force (ICCTF) and a recent meta-analysis.^12^^,^^32^ Due to methodological constraints RCIs were unavailable for the Rey CFT. In addition, to better align with seminal ­publications in the field not using RCI, a negative change of ≥1 z-score indicating a cognitive decline was also calculated.^20^

**Table 2.** Neuropsychological test results pre-operatively and one year post-operatively

				PRE-OP	POST-OP		PRE-OP	POST-OP	
Cognitive domain		Test variable	*n*	z-scores mean (SD)	z-scores mean (SD)	*P*-value	**z <** −**1.645**	**z <** −**1.645**	*P*-value
**Learning/Memory**	Visuo-spatial	BVMT-R Learning	126	−0.19 (1.25)	−0.05 (1.38)	0.183	15.9	15.1	0.88
		BVMT-R Del. Recall	126	0.06 (1.23)	−0.03 (1.37)	0.801	11.1	15.1	0.57
	Verbal	RAVLT Learning	126	0.02 (1.20)	−0.07 (1.38)	0.331	8.7	10.3	0.83
		RAVLT Del. recall	125	0.17 (1.09)	0.06 (1.27)	0.410	8.0	9.6	0.82
**Visuospat/percept.**	Speed	Trail Making Test A	85	0.06 (0.99)	−0.03 (0.83)	0.377	3.5	4.7	1.00
	Exec.	Rey CFT copy	109	−0.62 (1.03)	−0.61 (1.03)	0.298	21.8	20.9	1.00
**Language**	Visuo-perc.	Boston Naming Test	105	−0.75 (1.21)	−0.60 (1.42)	0.402	23.8	22.9	1.00
	Exec.	Phonemic fluency	110	−0.37 (1.36)	−0.40 (1.43)	0.621┼	15.5	20.9	0.38
	Speed	Semantic fluency	109	−0.46 (1.35)	−0.51 (1.69)	0.440	14.2	19.4	0.86
		CWIT 1	105	−0.60 (1.47)	−0.56 (1.16)	0.030^*^	13.3	20.0	0.27
		CWIT 2	101	−0.53 (1.15)	−0.87 (1.54)	0.071	17.8	22.8	0.48
**Executive**	Language	CWIT 3	105	−0.53 (1.34)	−0.61 (1.59)	0.046^*^	12.4	21.0	0.19
		CWIT 4	105	−0.62 (1.22)	−0.83 (1.76)	0.461	18.1	21.0	0.86
	Visuo-spatial	Trail Making Test B	85	−0.18 (1.17)	−0.34 (1.21)	0.547	9.4	12.9	0.63
	Attent.	Digit span backward	126	−0.50 (0.89)	−0.33 (0.88)	0.795	3.2	4.0	0.16
**Speed/Attention**	Attent.	Digit span forward	126	−0.34 (0.81)	−0.50 (0.88)	0.068	7.9	14.3	1.00
	Speed	Coding	86	−0.28 (0.81)	−0.19 (0.80)	0.569┼	4.7	4.7	1.00

Results for each cognitive test sorted by domains and sub-domains are presented as mean z-scores (SD) and proportions of patients classified as impaired (z < −1.645), pre-operatively and at one year post-operatively. Statistical tests assess both within-subject score changes and changes in impairment prevalence. Wilcoxon matched-pairs signed rank test, two-tailed was used due to the non-Gaussian distribution of variables, as determined by the Kolmogorov-Smirnov test. When normally distributed = ┼, paired t-tests were used. Proportions were examined using the Fisher’s exact test. Abbreviations: BVMT-R: Brief Visuospatial Memory Test—Revised; CWIT: D-KEFS Color Word Interference Test; RAVLT: Rey Auditory Verbal Learning Test; Rey CFT copy: Rey Complex Figure Test copying.

Heatmaps were generated in R (version 4.5.0; R Core Team, 2025) using the *ggplot2* package to visualize individual average changes in RCI scores across domains.

#### Predictive models

To assess regression assumptions, we examined correlations (>0.7) and conducted a linear regression to check multicollinearity using variance inflation factors (VIF < 5) and tolerance values. Dichotomous predictors were evaluated with crosstabulation. Linearity between continuous predictors and the logit was assessed using the Box-Tidwell procedure by including interaction terms with their natural logarithms. Univariable and multivariable logistic regression analyses were conducted to investigate predictors of reliable cognitive deterioration. The tests showing deterioration according to RCI in >20% of cases were analyzed.

Predictors representing demographic, functional, tumor-, and treatment-related factors were selected based on anticipated clinical relevance. To limit the risk of mass significance, only seven predictors were examined: preoperative age, preoperative Karnofsky Performance Status (KPS) score, preoperative tumor size (ml), presence of 1p19q-codeletion (yes/no), tumor grade (low/high), psychological health (preoperative HADS score >8 p in either anxiety or depression) and treatment with chemoradiotherapy (no/yes). The factors age and KPS are commonly used predictors and have been shown to be related to for example cognitive functioning and work ability.^7^^,^^33^ Tumor size was included since larger tumors involve and require treatment of larger areas of the brain, possibly leading to greater cognitive impairments and declines. 1p19q-codeletion was selected to account for known differences in tumor characteristics such as preferential location, growth rate, and velocity in oligodendroglioma.^1^ Treatment was represented by chemoradiotherapy (combined chemo- and radiotherapy) chosen as a typical guideline-based treatment, that has previously been found to impact cognitive functioning.^7^^,^^34^ Tumor grade was added due to its inherent link to growth rate and previously demonstrated association with cognitive outcome.^7^^,^^9^ Psychological health was assessed using HADS to examine how self-reported symptoms impact outcomes.

### Post-Hoc Analyses

To investigate the potential impact of radiotherapy type (protons vs photons) on cognition, we performed post-hoc Fisher’s exact tests for each cognitive domain.

### Heatmaps of Tumor Locations

To visually compare the differences in tumor location between patients with and without cognitive deterioration according to RCI, heatmaps of tumor locations were generated for each neuropsychological test. Tumor segmentations were spatially aligned by registration to the MNI-space as described previously.^35^^,^^36^ All images were inspected for registration errors and adjusted manually when necessary. In case of errors, we applied landmark registration with 3D Slicer.^37^ Tumor overlays were created by adding the registered segmentations for each group together. A colormap was applied on each tumor overlay separately, weighted by the maximal tumor overlap for each group. The Python programming language version 3.8.3 (Python Software Foundation) and 3D Slicer were utilized for creating heatmaps on tumor overlays.

## Results

### Participants

**Figure 1.**
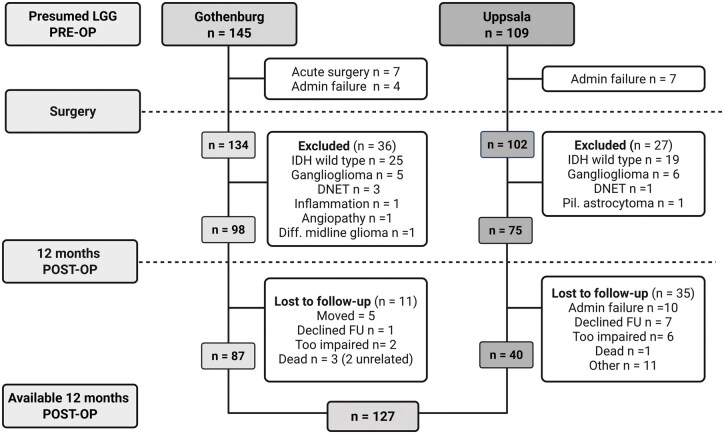
Flow chart of patient inclusion. Flow chart showing the selection and inclusion process of patients with IDH-mutated glioma who completed neuropsychological assessment pre-operatively and at one-year post-operatively. A total of 127 patients were included. Reasons for exclusion are shown at each step. Created in BioRender. Denes, A. (2025) https://BioRender.com/mrtg8p7

A total of 127 patients undergoing neuropsychological assessment before and at one year after surgery were included in the study. Details of the inclusion process are presented in Figure 1. One patient underwent an additional surgery (due to tumor remnant) before the one-year follow-up. No patients had tumor recurrence or progression during the first year. Patient, tumor, and treatment characteristics are presented in Table 1. Most patients underwent surgical resection (98%), received radiotherapy (82%), and chemotherapy (79%). Tumors were mostly WHO grade 2 (69%), astrocytomas (59%), and located in the frontal lobes (58%). The majority of patients (94%) were capable of normal daily activity (KPS ≥80) pre-operatively. Approximately one-third of the patients developed a new postoperative neurological deficit (transient or permanent) while 7% experienced a major permanent ­deficit (eg, dysphasia or paresis).

**Table 1.** Demographic, tumor, and treatment characteristics of included patients

Variable	Patients (*n* = 127)	Controls (*n* = 88)
Age pre-op, median (Q1-Q3)	41 (33-50)	40 (31-47)
Sex, male (%)	72 (56.7)	42 (48)
Education, mean years (range)	14 (7-22)	14 (8-21)
≤132, nr (%)	63 (50)	33 (38)
>132, nr (%)	64 (50)	55 (63)
Epilepsy at disease onset, nr (%)		
AED preop	86 (68)	
AED 12 months	96 (76)	
KPS ≥80, nr (%)		
Pre-op	119 (94)	
Post-op (3 months)	99 (81)	
HADS Score >8, nr (%)^a^		
Anxiety, preop	31 (27)	
Depression, preop	17 (15)	
Anxiety, 12 months	24 (21)	
Depression, 12 months	13 (11)	
Hemisphere, nr (%)		
Left	62 (49)	
Right	55 (43)	
Bilateral	10 (9)	
Tumor size in ml nr (%)		
Pre-op, Mdn (Q1-Q3)	45 (20-78)	
Extent of resection % Mdn (Q1-Q3)	92 (81-100)	
WHO 2021 grade nr (%)		
Astrocytoma 2	55 (43)	
Astrocytoma 3	13 (10)	
Astrocytoma 4	7 (6)	
Oligodendroglioma 2	33 (26)	
Oligodendroglioma 3	19 (15)	
Surgery type nr (%)		
Resection	124 (98)	
Biopsy	3 (2)	
Awake surgery (%)	46 (36)	
Postop deficits (%)		
Any deficit	43 (34)	
Permanent, major	9 (7)	
Radiotherapy before follow-up, nr (%)		
No	23 (18)	
Photon therapy	31 (24)	
Proton therapy	73 (57)	
Radiotherapy dose, Gy (fractions), nr (%)		
60 (2 × 30)	8 (6)	
59.4 (1.8 × 33)	26 (20)	
54 (1.8 × 30)	53 (42)	
50.4 (1.8 × 28)	16 (13)	
40.05 (2.67 × 15)	1 (1)	
Chemotherapy before follow-up, nr (%)		
No	27 (21)	
Temozolomide	61 (48)	
PCV	38 (30)	
Lomustine	1 (1)	
Time from chemotherapy to follow-up, weeks, Mdn (Q1-Q3)	8 (3-18)	

Summary of patient characteristics. Bilateral = bilateral involvement or evident infiltration into the opposite hemisphere.

a *n* = 115. Abbreviations: EOR: extent of resection; KPS: Karnofsky performance status; PCV: Procarbazine, Lomustine (CCNU), and Vincristine.

### Changes According to Published Normative Data

Table 2 presents the performance of patients in relation to published normative data, as well as the proportion of patients with impaired test results pre-operatively and at one year post-operatively in relation to published normative data. At group level, z-scores showed small differences between the pre-operative assessment and the one-year follow-up. Significant changes were observed in color naming (CWIT 1) and verbal inhibition (CWIT 3) when comparing medians, but not when comparing proportions of impaired patients. Pre-operatively, 59% of patients had at least one test score classified as impaired, and 39% had two or more impaired scores. The corresponding percentage at one year was 62% for one score, and 44% for two or more scores. When applying the z < −1.5 SD cutoff for classification of impairment, 71% of patients in our cohort had at least one impaired score, 28% had three or more, and 12% had five or more impairments pre-operatively. The Monte Carlo simulation revealed that both the preoperative and postoperative proportions were substantially higher than expected by chance (*P *< 0.001).^29^ Test results presented as group level raw values for the pre-operative and one-year assessments are available in Table S2.

### Changes According to RCI

**Figure 2.**
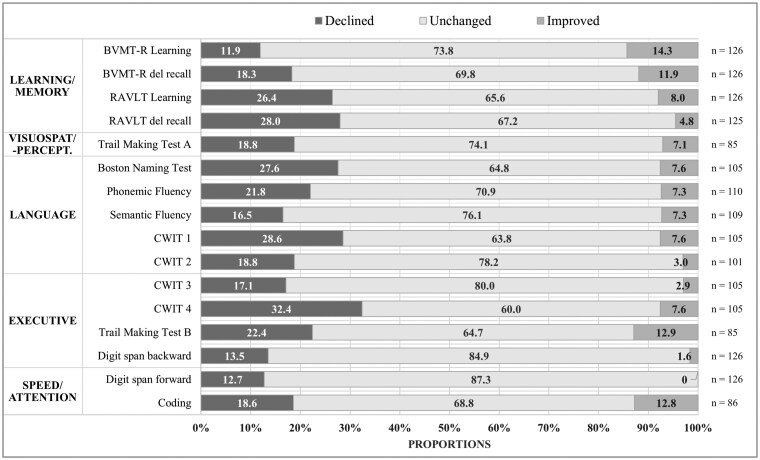
Proportion of patients showing reliably changed results in each test variable. Bar chart illustrating the proportion of patients with significant cognitive changes presented per neuropsychological test, based on Reliable Change Indices (RCI). Abbreviations: BVMT-R: Brief Visuospatial Memory Test—Revised; CWIT: Color Word Interference Test; RAVLT: Rey Auditory Verbal Learning Test; Rey CFT: Rey Complex Figure Test.

Proportions of reliably changed patients per test variable according to RCI are presented in Figure 2. Proportions of significant declines varied from 11.9% to 32.4% with the most prominent declines seen in the test of verbal inhibition/flexibility (CWIT 4, 32.4%) and naming speed (CWIT 1, 28.6%). Prominent declines were also seen in verbal learning and memory (RAVLT learning 26.4%, RAVLT memory 28.0%), object naming (BNT, 27.6%), non-verbal flexibility (TMT B, 22.4%), and verbal fluency (Phonemic fluency 21.8%). Improvements were seen in 0-14.3% depending on the test, with the largest improvements seen in BVMT-R learning, TMT B, Coding, and BVMT-R recall. Proportion of domain-level declines were as follows: executive (24.1%), learning/memory (23.2%), language (21.1%), visuospatial-/perceptual (17.3%), and speed/attention (14.4%).

Changes in results according to the difference between pre- and post-operative scores, both as Δz scores and as RCIs, are presented in Table S3.

Heatmaps of individual RCI-scores per domain can be found in Figure S1.

### Regression Models

Table 3 presents results from the logistic regression models for the tests showing the most declines (>20%). In the multivariable models predicting declines in single tests, age was a significant predictor in three of the models (CWIT 4, *P *= 0.002; CWIT 1, *P *= 0.033; RAVLT delayed recall *P *= 0.045), and chemoradiotherapy was a significant predictor in two of the models (CWIT 4, *P *= 0.032; CWIT 1, *P *= 0.021). For the phonemic fluency, the criteria for regression analysis were not met for the chemoradiotherapy variable, as all patients with significant impairments had received this treatment. In the domain-level regression analyses, age was an independent predictor for declines in learning/memory (*P *= 0.007) and executive functioning (*P *= 0.013). Chemoradiotherapy was an independent predictor for declines in language (*P *= 0.037) and executive functioning (*P *= 0.033). Since the focus of this paper was on changes following treatment, and identifying risk factors for cognitive decline, improvements were not analyzed further.

**Table 3.** Logistic regression models for identifying predictors for cognitive decline according to reliable change indices

		Covariate	**Univariable** **OR (95% CI)**	*P*-value	**Multivariable,** **OR (95% CI)**	*P*-value
**Learning/Memory**	**Verbal**	**RAVLT del. Recall**				
		Age	1.04 (1.00-1.08)	0.049^*^	1.05 (1.00-1.09)	0.045^*^
		KPS pre-operative	0.97 (0.93-1.01)	0.146	0.98 (0.93-1.04)	0.548
		Tumor size	0.99 (0.99-1.01)	0.620	1.00 (0.98-1.01)	0.533
		1p19q codeletion	0.76 (0.34-1.72)	0.512	0.65 (0.25-1.67)	0.368
		High grade	0.89 (0.38-2.10)	0.792	1.07 (0.39-2.93)	0.897
		HADS >8 p preop	1.30 (0.53-3.20)	0.562	1.36 (0.53-3.49)	0.522
		Chemoradiotherapy	1.63 (0.56-4.77)	0.369	1.51 (0.45-5.07)	0.509
**Language**	**Visuoperceptual**	**Boston naming test**				
		Age	1.02 (0.98-1.06)	0.260	1.03 (0.99-1.08)	0.173
		KPS pre-operative	1.00 (0.95-1.05)	0.945	1.01 (0.96-1.07)	0.639
		Tumor size	1.00 (0.99-1.01)	0.942	1.00 (0.99-1.02)	0.650
		1p19q codeletion	0.72 (0.30-1.76)	0.477	0.66 (0.25-1.74)	0.397
		High grade	0.73 (0.29-1.82)	0.498	0.78 (0.28-2.16)	0.629
		HADS >8 p preop	1.40 (0.54-3.62)	0.487	1.45 (0.53-3.93)	0.468
		Chemoradiotherapy	1.67 (0.51-5.48)	0.400	1.69 (0.46-6.26)	0.430
	**Executive**	**Phonemic fluency**				
		Age	1.02 (0.98-1.07)	0.298	1.02 (0.98-1.07)	0.374
		KPS pre-operative	0.99 (0.94-1.04)	0.745	1.00 (0.94-1.07)	0.905
		Tumor size	1.00 (0.99-1.01)	0.959	1.00 (0.99-1.01)	0.877
		1p19q codeletion	0.98 (0.38-2.52)	0.972	0.81 (0.29-2.27)	0.687
		High grade	1.59 (0.62-4.08)	0.331	1.95 (0.67-5.65)	0.220
		HADS >8 p preop	1.32 (0.47-3.65)	0.599	1.47 (0.51-4.25)	0.474
		Chemoradiotherapy	N.A.	N.A.	N.A.	N.A.
	**Speed**	**CWIT 1**				
		Age	1.04 (1.00-1.08)	0.043^*^	1.04 (1.00-1.09)	0.033^*^
		KPS pre-operative	0.98 (0.93-1.02)	0.328	1.00 (0.94-1.04)	0.932
		Tumor size	1.00 (0.99-1.01)	0.619	0.99 (0.98-1.00)	0.203
		1p19q codeletion	2.21 (0.98-4.98)	0.055	2.44 (0.92-6.46)	0.073
		High grade	2.65 (1.14-6.14)	0.023^*^	2.71 (0.70-7.55)	0.057
		HADS >8 p preop	2.27 (0.88-5.89)	0.091	2.16 (0.72-6.50)	0.172
		Chemoradiotherapy	3.97 (1.08-14.57)	0.038^*^	6.05 (1.32-27.80)	0.021^*^
**Executive**	**Language**	**CWIT 4**				
		Age	1.06 (1.02-1.10)	0.003^*^	1.08 (1.03-1.13)	0.002^*^
		KPS pre-operative	0.95 (0.91-1.00)	0.040^*^	0.99 (0.93-1.05)	0.632
		Tumor size	1.01 (1.00-1.02)	0.245	1.00 (0.99-1.01)	0.751
		1p19q codeletion	1.01 (0.49-2.37)	0.862	0.92 (0.35-2.41)	0.867
		High grade	2.59 (1.12-5.97)	0.025^*^	2.64 (0.98-7.13)	0.056
		HADS >8 p preop	1.26 (0.49-3.26)	0.629	1.19 (0.40-3.60)	0.754
		Chemoradiotherapy	4.58 (1.25-16.81)	0.022^*^	5.60 (1.16-26.99)	0.032^*^
	**Visuospatial**	**Trail making test B**				
		Age	1.01 (0.96-1.05)	0.744	1.00 (0.95-1.06)	0.991
		KPS pre-operative	1.01 (0.94-1.01)	0.841	0.98 (0.89-1.01)	0.639
		Tumor size	0.99 (0.98-1.01)	0.233	0.99 (0.97-1.01)	0.367
		1p19q codeletion	0.87 (0.31-2.49)	0.796	0.79 (0.23-2.66)	0.698
		High grade	0.44 (0.13-1.47)	0.180	0.44 (0.11-1.71)	0.234
		HADS >8 p preop	2.27 (0.70-7.38)	0.172	2.20 (0.62-7.84)	0.224
		Chemoradiotherapy	0.69 (0.21-2.25)	0.535	1.23 (0.28-5.40)	0.781

Univariable and multivariable logistic regression results for the cognitive tests showing >20% decline according to RCI. Predictors included age (per year), preoperative KPS (0-100), tumor size (ml), tumor laterality (right/left), presence of 1p19q-codeletion (no/yes), high grade tumor (grade 2 vs 3-4) (no/yes), HADS score >8 p preoperatively (in either subscale) (no/yes), and combined treatment with chemo- and radiotherapy (chemoradiotherapy) (no/yes). Tests are presented domain-wise. Odds ratios (95% CI) and *P*-values are reported. Significant results (*P* < 0.05) are marked with * Abbreviations: CWIT: D-KEFS Color Word Interference Test; RAVLT del recall: Rey Auditory Verbal Learning Test delayed recall; HADS: Hospital Anxiety and Depression Scale.

### Post-Hoc Analysis

The post-hoc analysis on type of radiotherapy (protons vs photons) showed no significant results: learning/memory *P *= 0.459; language *P *= 0.481; speed/attention *P *= 0.890; executive *P *= 0.115; visuospatial/perceptual *P *= 1.000.

### Heatmaps

**Figure 3.**
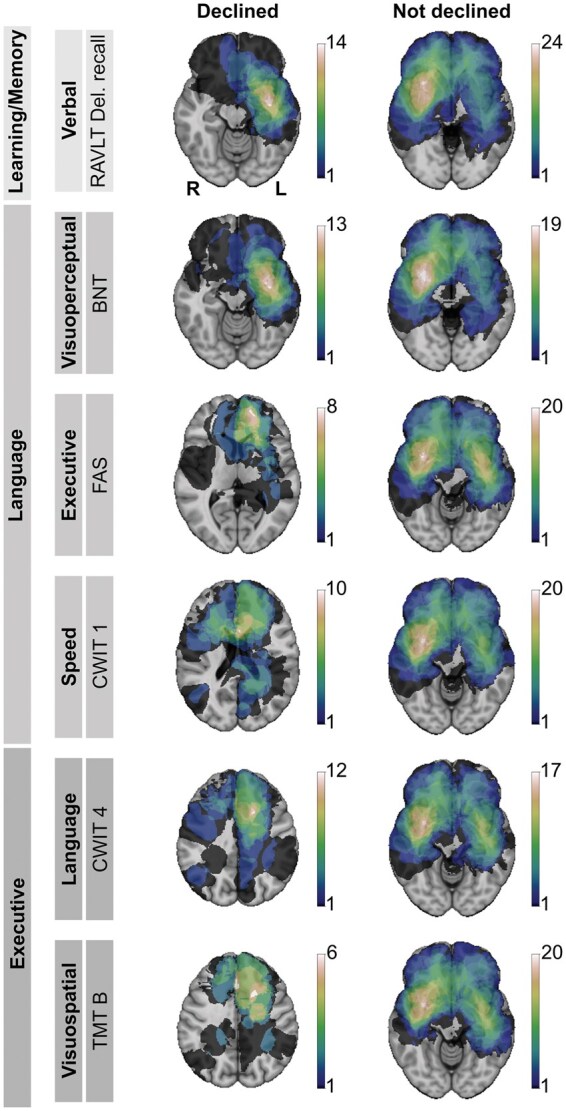
Heatmaps of tumor locations for tests showing the most cognitive declines. Heatmaps visualizing tumor locations in patients with versus without reliable cognitive declines (based on RCI) for the six neuropsychological tests with the highest decline rates. Color intensity reflects the spatial overlap of tumors, with warmer colors indicating greater overlap. Hemispheres are mirrored and tests are sorted by domains and subdomains. Abbreviations: BNT: Boston Naming Test; CWIT: Color Word Interference Test; RAVLT del recall: Rey Auditory Verbal Learning Test, delayed recall; TMT B: Trail Making Test B.

Heatmaps illustrating tumor locations for patients with significant declines in the six tests showing the most frequent declines (>20%) according to RCIs are presented for patients with versus without significant cognitive declines in Figure 3. As illustrated, a left sided hotspot tumor localization was found in the test showing most declines, and a hot spot in the right hemisphere was displayed in cases of intact or improved cognition. Corresponding figures showing tumor locations for patients with significant declines when organized domain-wise are presented in Figure S2.

More detailed heatmaps illustrating the same individual tests with most declines (>20%) are shown in Figures S3-S8. Figures S9-S16 display the same type of heatmaps for the other tests with less than 20% cognitive declines. Detailed heatmaps illustrating tumor location of patients with the most prominent declines within a domain (average RCI-score) are presented in Figures S17-S21.

## Discussion

In this longitudinal study of patients with a first-time diagnosis of *IDH* mutated glioma, cognitive impairments were common already before treatment, and spanning multiple domains. According to RCI, a high proportion of declines (>20% of patients) within several cognitive domains were seen at one year after surgery. These declines were most prominent in executive functioning, learning/memory, and language. Older age and treatment with chemoradiotherapy were independent predictors of cognitive decline.

Two larger studies on cognitive changes following surgery in patients with *IDH* mutated grade 2 gliomas using comprehensive neuropsychological assessments have recently been published.^23^^,^^28^ Of note, the two studies are from the same research group, and based on overlapping patient cohorts. In contrast to our cohort, neuropsychological evaluations were done before oncological treatment.^23^^,^^28^ In the first study, by Lemaitre et al., 55.4% of patients had at least one impaired score pre-operatively (cut off z < −1.645).^23^ This result is comparable to our pre-operative results (59.1%). Lemaitre et al. did not report proportions with impairment at one year, but our data showed that the proportion of patients with impairment only slightly increased at one year (62.2%). Compared to the second study by Ng et al., our patients more commonly presented with impairments already before surgery, with object naming showing the largest difference between the cohorts (4.6% vs 23.8%), followed by TMT B (3.1% vs 9.4%), semantic fluency (8.0% vs 14.2%) and phonemic fluency (10.7% vs 15.5%).^28^ This discrepancy likely reflects differences in case selection and is not surprising given the population-based setting in our study, whereas their study focused on outcome following awake surgery at a highly specialized centre.

Another seminal work on patients with IDH-mutated gliomas used −1.5 SD as cut off and found that 77% of patients had at least one impaired score, 25% had three or more impairments, and 9% had five or more impairments.^9^ Applying this cut-off in our cohort yielded impairment rates of 71%, 28%, and 12%, respectively, which were comparable to their preoperative findings.

The largest increase in impairments following treatment, meaning the largest proportion of patients with results indicative of impairment, was seen in tests of executive functioning. Particularly the verbal interference test (CWIT 3), where impairments increased from 12.4% to 21.0%. Similarly, a large increase was seen for the color naming speed (CWIT 1). Taken together, these results support the vulnerability of verbal executive processing and speed to changes following multimodal treatment.

Clinically, patients often struggle when experiencing substantial declines, even when their scores remain within the normal range, meaning they are not classified as impaired. This is especially relevant for individuals whose occupations require high cognitive functioning, where such changes can significantly impact daily life and professional performance. While group-level performance may appear stable, measuring within-individual changes over time often provides more meaningful insights. Using a Δz > 1 criterion is one way to study changes.^20^^,^^23^ By using this criterion, we observed mostly similar proportions of declines as Lemaitre et al., but found substantial difference in the executive test   TMT B, with 25% declines in our group compared to 11% in their group.^23^ However, Lemaitre et al.included a more selective cohort limited to WHO grade 2 gliomas and assessed outcomes three months postoperatively, prior to oncological treatment. Differences in findings may reflect the potential impact of oncological treatment on cognitive outcomes, but can also be related to different tumor location or methodological differences, such as more widespread use of cognitive mapping (beyond motor and language) in their cohort.

Assessing cognitive changes requires caution, and relying solely on normative data from a single testing can be misleading due to practice effects. In the present study, despite applying a stricter z-score threshold for decline in the RCI compared to the Δ z > 1 criterion, the RCI sometimes showed a larger proportion of patients as significantly declined and smaller proportion with improvements, suggesting the presence of learning effects. In our control group, improvements were seen across all test variables. For example, controls improved an average of 1.3 words in the object naming test (BNT). Previous studies have shown that practice effects can be long-lasting, both with repeated testing and after only a single prior exposure to the test.^38^ This highlights the value of using RCIs in detecting cognitive changes. As with the use of Δz > 1, our RCI results revealed significant declines in the TMT B and CWIT 4, indicating that executive functions were the most affected functions. This was also supported by our domain level RCI analyses. Given the common frontal lobe involvement of *IDH* mutated gliomas this is not unexpected.^39^ However, we also saw significant declines (>20%) in individual tests of verbal speed, memory and language, and domain-wise in executive functioning, learning/memory, and language following treatment. These results align well with a meta-analysis on cognitive functioning following multimodal treatment in patients with various types of gliomas.^12^ Overall, declines outweighed improvements in our sample according to the RCI. However, in the BVMT-R, improvements were comparable or even greater than declines. For the Coding and TMT B tests, more than 10% also showed improvements, though declines were clearly more frequent, especially for the TMT B.

Avoiding cognitive decline is a key goal following treatment. Our regression analyses revealed that older age and treatment with chemoradiotherapy were both associated with an increased risk of decline. Age was a significant predictor of decline in the domains learning/memory and executive functioning. It is reasonable to assume that older age could be associated with a prolonged convalescence, and possibly less plasticity potential.^25^ Age-related declines in learning and memory, as well as executive functioning, are well established.^7^^,^^40–43^ The impact of age on change scores is less studied, although documented in both the RAVLT and CWIT.^41^ Chemoradiotherapy was a significant predictor for decline in language and executive functioning. This indicates that this treatment may negatively affect cognitive functioning already at an early time point during the disease trajectory, particularly the performance in tasks involving verbal processing and executive functioning.^7^^,^^12^ In a recently published long-term follow-up study of patients with oligodendroglioma by Boele et al., all cognitive tests showed higher impairment rates than in our cohort (using the same −1.5 SD criterion).^34^ For example, impairments in TMT A and TMT B were 18% and 21% in their study, compared with 6% and 13% in our full cohort and 12% and 11% in patients with oligodendroglioma only. They also found time since diagnosis to be a significant predictor of impairment, supporting the notion of progressive decline over time. In addition, radiotherapy was associated with worse cognitive performance, aligned with our own findings. Negative effects related to chemoradiotherapy have also been shown in another recent study by Lanman et al., who reported that both chemotherapy and radiotherapy were associated with long-term cognitive impairment in patients with IDH mutated glioma, suggesting that treatment-related neurotoxicity may contribute to cognitive decline that can persist over time.^7^

In our study, the follow-up period was limited to one year, which is insufficient to capture long-term cognitive sequelae. Although it remains unclear whether the observed declines were transient or persisted over time, we consider it most likely that the changes were related to treatment, as patients were tested before and after treatment during a stable disease phase. Worsening that occurs later may be driven by multiple factors with uncertain relative contributions. A landmark long-term follow-up study by Douw et al. on patients with low-grade gliomas comparing patients who received radiotherapy (without chemotherapy) versus those who received no adjuvant treatment showed cognitive declines only in the group receiving radiotherapy.^44^ In a systematic review, the authors concluded that radiotherapy may have long-term negative effects on cognitive function, although the evidence is insufficient to determine the extent of the risk.^45^ Both this review and the one by Koutsarnakis et al. revealed mixed findings, likely influenced by confounders such as the use of previous WHO classifications lacking *IDH* status information and subsequently the inclusion of mixed cohorts of *IDH* mutant and *IDH* wild-type tumors.^45^^,^^46^ In a cross-sectional long-term study, Klein et al. observed no cognitive impairment (>2 SD criterion) after radiotherapy with fraction doses below 2 Gy.^47^ Although they did not study individual changes, it is possible that early cognitive decline can in some cases be transient, with initial improvement before a later onset of long-term impairment.

New targeted treatments for *IDH* mutated gliomas are emerging, such as *IDH* inhibitors, showing to prolong the time to progression with limited toxicities.^48^ This offers promising prospects for future treatment. In the present study, we presented data on cognitive functioning the first year after initial treatment according to the current treatment standard, prior to the widespread clinical introduction of *IDH* inhibitors. Our regression models indicated that age and chemoradiotherapy were independently associated with cognitive decline in several tests and domains. Our models, however, were limited and factors such as radiation dose, and type of chemotherapy were not analyzed. The post-hoc analysis on type of radiotherapy showed no difference between radiotherapy treatment using photons or protons.

Additional factors may have affected our results. Almost all of our patients were treated with the concept of maximal safe resection and many were treated with both resective surgery and oncological treatment. Since transient deficits are commonly seen postoperatively, we speculate if a synergistic effect can occur, as chemoradiotherapy is often initiated before full recovery from surgery in our setting. As mentioned, radiotherapy has been studied independently in glioma, but the effect of chemotherapy has not been examined in isolation.^44^ Preclinical studies have for example, demonstrated hippocampal damage and memory impairment after vincristine exposure.^49^ Since chemoradiation is the standard of care, disentangling the specific effects of chemotherapy is challenging. Evidence from other cancers shows that chemotherapy can cause cognitive changes, but may on the other hand stabilize cognition through tumor control.^21^ Fatigue related to chemotherapy may also contribute, making some impairments temporary. We consider this factor to have less impact, since longer-term data show greater, not fewer, impairments.^34^ Timing of treatment was not standardized in our study, but the median time since last chemotherapy dose was approximately two months. For patients still receiving treatment, assessments were scheduled when fatigue was minimal, usually just before the next chemotherapy dose. Still, the finding that chemoradiotherapy is associated with cognitive decline at this time point is important as treatment is one of the few potentially modifiable factors. A substantial proportion of patients with *IDH* mutated gliomas may live for decades and there is a risk for long-term cognitive consequences.^50^^,^^51^ Although we acknowledge a proven survival benefit of combination therapy, we are not convinced that the optimal timeframe for initiating such treatment is as narrow as proposed in the current guidelines.^1^ Early oncological treatment could be considered for high-risk patients following thorough, individualized consideration. Guidelines for what is high-risk may need revision not only due to emerging treatments, but also because the previous risk assessment based upon mainly age >40 years and (any) tumor remnant might be too crude.^1^ Our findings may provide useful baseline data for potential future treatment decisions. By identifying risk factors treatment can be tailored to patient risk profiles to avoid cognitive deterioration. Future research should refine predictive models, assess long-term effects in longitudinal studies, and explore interventions to prevent cognitive impairments.

Strengths of this study include the use of longitudinal data beginning prior to any treatment, a large and unselected cohort of patients with histopathologically verified *IDH*-mutated gliomas classified according to the latest WHO classification, and treatment administered in line with current standards prior to the clinical introduction of *IDH* inhibitors. A key strength is also the use of well-established neuropsychological tests, and comparison to both published normative data as well as a matched control group with the use of RCI.

Limitations include reduced sample size due to test differences across centers, limited visuo-spatial measures, and restricted verbal testing for non-Swedish speakers. COVID-19 contributed to dropout, and the sample size limited the number of predictors in the regression models. Another limitation is the lack of systematic postoperative follow-up, which would have helped to clarify the effects of surgery and oncological treatment. As chemotherapy and radiotherapy were analyzed only in combination, their specific effects on cognition were not possible to disentangle. We included seven patients with focal grade 4 transformation. All were clinically stable and showed no greater impairment or decline than the rest of the group. Further, by using many cognitive tests the likelihood of deviations by chance increase. To address this, we applied more stringent criteria for defining impairment (z <−1.645 compared to the commonly used −1.5 SD). In addition, we performed Monte Carlo simulations to assess whether impairment were greater than expected by chance and found that they were.^29^ Also, if we would have large random variation in the RCI, we would expect it to be seen both in improvements and declines, but we found that declines were far more prominent than improvements.

## Conclusion

Our study found that cognitive impairments were common across several cognitive domains even before treatment. Although group-level results remained relatively stable after treatment, within-individual analyses revealed notable declines in executive functioning, learning/memory, and language. This underscores the importance of longitudinal monitoring at an individual level. Regression analyses identified older age and chemoradiotherapy as possible risk factors for declines in specific functions. The association between chemoradiotherapy and cognitive decline reinforces concerns about its potential cognitive effects, particularly as this is a modifiable variable.

## Supplementary Material

noaf263_Supplementary_Data

## Data Availability

Due to the nature of this research, participants did not agree to public sharing of their data. Hence, non-identifiable group level data can be shared and is available from the corresponding author upon reasonable request.

## Previous Dissemination

Preliminary results of this study have previously been presented at the Society of Neuro-Oncology meeting in Vancouver 2023, and at the Scandinavian Society of Neuro-oncology symposium in Gothenburg 2024.
