# Supplementary material for: Reliable cognitive changes the first year following guideline-based treatment of isocitrate dehydrogenase mutated gliomas: A longitudinal multicenter study
Source: Neuro Oncol. 2025 Nov 9;28(3):704–16. doi: 10.1093/neuonc/noaf263 (PMC13070499; doi:10.1093/neuonc/noaf263)
Supplement: noaf263_Supplementary_Data [file noaf263_supplementary_data.zip › CaptionsLegendsSupplementaryFigures1to21REV.docx]

**Supplementary figure 1.****Heatmap of individual RCI-scores across domains.**

Heat map illustrating individual average changes in RCI scores per domain (columns) with each row representing one patient. Green = improvement, red = decline, grey = missing. The column to the right show patients’ mean score across all domains. RCI ±1.65 indicate significance.

**Supplementary figure** **2**

**Heatmaps of tumor locations organized per cognitive domain.**

Heatmaps visualizing tumor locations in patients with versus without reliable cognitive declines per cognitive domain, based on average RCI-scores within each domain. Color intensity reflects the spatial overlap of tumors, with warmer colors indicating greater overlap. Hemispheres are mirrored.

Supplementary figure 3

Heatmaps of tumor locations in patients with significant declined result in RAVLT delayed recall (verbal memory). A. shows patients with significant declines, and B. shows patients without significant declines. Color intensity reflects the spatial overlap of tumors, with warmer colors indicating greater overlap. Hemispheres are mirrored.

**Supplementary figure 4**

Heatmaps of tumor locations in patients with significant declined result in Boston Naming test (BNT). A. shows patients with significant declines, and B. shows patients without significant declines. Color intensity reflects the spatial overlap of tumors, with warmer colors indicating greater overlap. Hemispheres are mirrored.

Supplementary figure 5

Heatmaps of tumor locations in patients with significant declined result in D-KEFS Phonemic Fluency test. A. shows patients with significant declines, and B. shows patients without significant declines. Color intensity reflects the spatial overlap of tumors, with warmer colors indicating greater overlap. Hemispheres are mirrored.

Supplementary figure 6

Heatmaps of tumor locations in patients with significant declined result in D-KEFS Color Word Interference Test 1 (CWIT 1). A. shows patients with significant declines, and B. shows patients without significant declines. Color intensity reflects the spatial overlap of tumors, with warmer colors indicating greater overlap. Hemispheres are mirrored.

Supplementary figure 7

Heatmaps of tumor locations in patients with significant declined result in D-KEFS Color Word Interference Test 4 (CWIT 4). A. shows patients with significant declines, and B. shows patients without significant declines. Color intensity reflects the spatial overlap of tumors, with warmer colors indicating greater overlap. Hemispheres are mirrored

Supplementary figure 8

Heatmaps of tumor locations in patients with significant declined result in the Trail Making Test B (TMT B). A. shows patients with significant declines, and B. shows patients without significant declines. Color intensity reflects the spatial overlap of tumors, with warmer colors indicating greater overlap. Hemispheres are mirrored.

Supplementary figure 9

Heatmaps of tumor locations in patients with significant declined results in the Brief Visuo-Spatial Memory Test – Revised delayed recall (BVMT-R del. recall). A. shows patients with significant declines, and B. shows patients without significant declines. Color intensity reflects the spatial overlap of tumors, with warmer colors indicating greater overlap. Hemispheres are mirrored.

Supplementary figure 10

Heatmaps of tumor locations in patients with significant declined result in the Trail Making Test A (TMT A). A. shows patients with significant declines, and B. shows patients without significant declines. Color intensity reflects the spatial overlap of tumors, with warmer colors indicating greater overlap. Hemispheres are mirrored.

Supplementary figure 11

Heatmaps of tumor locations in patients with significant declined result in the D-KEFS Semantic Fluency, animals. A. shows patients with significant declines, and B. shows patients without significant declines. Color intensity reflects the spatial overlap of tumors, with warmer colors indicating greater overlap. Hemispheres are mirrored.

Supplementary figure 12

Heatmaps of tumor locations in patients with significant declined result in the D-KEFS Color Word Interference Test 2 (CWIT 2). A. shows patients with significant declines, and B. shows patients without significant declines. Color intensity reflects the spatial overlap of tumors, with warmer colors indicating greater overlap. Hemispheres are mirrored.

Supplementary figure 13

Heatmaps of tumor locations in patients with significant declined result in the D-KEFS Color Word Interference Test 3 (CWIT 3). A. shows patients with significant declines, and B. shows patients without significant declines. Color intensity reflects the spatial overlap of tumors, with warmer colors indicating greater overlap. Hemispheres are mirrored.

Supplementary figure 14

Heatmaps of tumor locations in patients with significant declined result in the WAIS IV Digit span backward. A. shows patients with significant declines, and B. shows patients without significant declines. Color intensity reflects the spatial overlap of tumors, with warmer colors indicating greater overlap. Hemispheres are mirrored.

Supplementary figure 15

Heatmaps of tumor locations in patients with significant declined result in the WAIS IV Digit span forward. A. shows patients with significant declines, and B. shows patients without significant declines. Color intensity reflects the spatial overlap of tumors, with warmer colors indicating greater overlap. Hemispheres are mirrored.

Supplementary figure 16

Heatmaps of tumor locations in patients with significant declined results in the WAIS IV Coding. A. shows patients with significant declines, and B. shows patients without significant declines. Color intensity reflects the spatial overlap of tumors, with warmer colors indicating greater overlap. Hemispheres are mirrored.

Supplementary figure 17

Heatmaps of tumor locations in patients with an average significant decline in the learning and memory domain. A. shows patients with significant declines, and B. shows patients without significant declines. Color intensity reflects the spatial overlap of tumors, with warmer colors indicating greater overlap. Hemispheres are mirrored.

**Supplementary figure 18**

Heatmaps of tumor locations in patients with an average significant decline in the visuo-spatial and perceptual domain. A. shows patients with significant declines, and B. shows patients without significant declines. Color intensity reflects the spatial overlap of tumors, with warmer colors indicating greater overlap. Hemispheres are mirrored.

Supplementary figure 19

Heatmaps of tumor locations in patients with an average significant decline in the language domain. A. shows patients with significant declines, and B. shows patients without significant declines. Color intensity reflects the spatial overlap of tumors, with warmer colors indicating greater overlap. Hemispheres are mirrored.

Supplementary figure 20

Heatmaps of tumor locations in patients with an average significant decline in the executive domain. A. shows patients with significant declines, and B. shows patients without significant declines. Color intensity reflects the spatial overlap of tumors, with warmer colors indicating greater overlap. Hemispheres are mirrored.

Supplementary figure 21

Heatmaps of tumor locations in patients with an average significant decline in the speed and attention domain. A. shows patients with significant declines, and B. shows patients without significant declines. Color intensity reflects the spatial overlap of tumors, with warmer colors indicating greater overlap. Hemispheres are mirrored.
