# Supplementary material for: Reliable cognitive changes the first year following guideline-based treatment of isocitrate dehydrogenase mutated gliomas: A longitudinal multicenter study
Source: Neuro Oncol. 2025 Nov 9;28(3):704–16. doi: 10.1093/neuonc/noaf263 (PMC13070499; doi:10.1093/neuonc/noaf263)
Supplement: noaf263_Supplementary_Data [file noaf263_supplementary_data.zip › New supplementary material.docx]

**New supplementary material**

**Reliable change index**This is a description of the adjusted regression-based Reliable Change Index used in the manuscript. This model was originally presented as Model 10 in Maassen, Bossema, and Brand (2009):

- $D_{i}$ The average difference in a test variable among patients calculated as the result at 12 months minus the result at 0 months (post - pre)
- $\bar{D}_{C}$ The average difference in a test variable among controls (post – pre)
- $S_{Y}$ Standard deviation and $S_{Y}^{2}$ the variance of controls at 12 months
- $S_{X}$ Standard deviation and $S_{X}^{2}$ the variance of controls at 0 months
- $X_{i}$ The individuals test score at 0 months
- $\bar{X}_{C}$ The average test score in controls at 0 months
- $r_{XY}$The product-moment correlation coefficient for the relationship between the controls’ scores at 0 and 12 months

Reliable Change Index (RCI) estimates whether an individual’s change in test performance exceeds what would be expected due to measurement error, natural variability, or practice effects. It accounts for test–retest reliability, individual baseline scores, and the variability observed in a control group tested at the same intervals. By incorporating the average change and standard deviations from a matched healthy control group, the method adjusts for expected improvements due to familiarity with the tests. It also considers regression to the mean by including in the individual’s initial (preoperative) performance. This produces a more accurate estimate of whether a cognitive change reflects a true deviation from expected patterns, rather than random fluctuation, and enables detection of meaningful individual-level changes in the test results.

**Reference:** Maassen, G. H., Bossema, E., & Brand, N. (2009). Reliable change and practice effects: outcomes of various indices compared. *Journal of Clinical and Experimental Neuropsychology, 31*, 339-352.
