# Supplementary material for: Reliable cognitive changes the first year following guideline-based treatment of isocitrate dehydrogenase mutated gliomas: A longitudinal multicenter study
Source: Neuro Oncol. 2025 Nov 9;28(3):704–16. doi: 10.1093/neuonc/noaf263 (PMC13070499; doi:10.1093/neuonc/noaf263)
Supplement: noaf263_Supplementary_Data [file noaf263_supplementary_data.zip › NewSupplementaryTables.docx]

**Supplementary material**

**Supplementary table 1. Test description**

| **Test** | **Description** |
| --- | --- |
| Boston Naming Test (BNT) | A 60-item confrontational picture naming test. |
| D-KEFS Verbal Fluency; semantic fluency, and phonemic fluency | Semantic: generate as many words as possible from the semantic category *animals* within one minute. Phonemic: generate words starting with a specific letter (F, A, S), without using names or numbers, for one minute each. Both measure verbal fluency and speed and the phonemic also executive functioning. |
| Rey Auditory Verbal  Learning Test (RAVLT) | A 15-item list of words is read five times, with a request to recall as many words as possibly each time. A delayed recall is performed after 30 minutes. Measures verbal learning and memory. |
| Rey Complex figure,  (RCFT) copying | Draw a complex neutral figure while looking at a model. Tests visuospatial construction (executive functioning). |
| Brief Visuospatial Memory Test-Revised (BVMT-R) | A set of six figures is presented three times x 10 seconds, after each time the participant is asked to draw the figures as similar as possible. After 25 minutes a delayed free recall is performed. Measures visuo-spatial learning and memory. |
| Trail Making Test, (TMT)  A and B | A, the participant is asked to connect circled numbers on time (visual attention and process speed). In task B, the participant is asked to connect numbers and letters alternatively in a specific order, adding a measure of mental (non-verbal) flexibility (executive functioning). |
| D-KEFS Color-Word Interference Test (CWIT) | All subtests should be performed as rapidly as possible.  CWIT 1. Naming colors of boxes, measures naming speed.  CWIT 2. Reading color names, measures reading speed.  CWIT 3. Naming the color of incongruently colored words, measures verbal speed and inhibition/interference (executive functioning).  CWIT 4. Same as 3 but alternating with reading the written words, measures cognitive flexibility (executive functioning). |
| WAIS-IV, Digit Span | A sequence of numbers is read of increasingly length.  Forward: directly repeat the numbers in order. Measures short-term auditory memory and concentration Backward: numbers are presented and should be repeated in the reverse order. Mesuress auditory working memory (executive functioning). |
| WAIS IV, Coding | Match numbers and symbols based on a visible key-code and draw as many correct symbols as possible in pre-printed boxes within two minutes. Measures mental speed, sustained attention and visuo-motor co-ordination. |
| **Self-assessment** | **Description** |
| The Hospital Anxiety and Depression Scale (HADS) | HADS consists of 14 items, seven items for the anxiety subscale and seven for the depression subscale. Response options range from 0-3. A score > 8 points was considered symptomatic. |

**References**

1. Benedict R. Brief visuospatial memory test - revised: Professional manual Lutz, FL: Psychological Assessment Resources, Inc; 1997.

2. Stricker NH, Christianson TJ, Lundt ES, Alden EC, Machulda MM, Fields JA, et al. Mayo Normative Studies: Regression-Based Normative Data for the Auditory Verbal Learning Test for Ages 30-91 Years and the Importance of Adjusting for Sex. J Int Neuropsychol Soc. 2021;27(3):211-26.

3. Tombaugh TN. Trail Making Test A and B: normative data stratified by age and education. Arch Clin Neuropsychol. 2004;19(2):203-14.

4. Espenes J, Lorentzen IM, Eliassen IV, Hessen E, Waterloo K, Timón-Reina S, et al. Regression-based normative data for the D-KEFS Color-Word Interference Test in Norwegian adults ages 20-85. Clin Neuropsychol. 2024;38(5):1227-55.

5. Delis DC, Kaplan, E., & Kramer, J. H. Delis-Kaplan Executive Function System (D–KEFS): APA PsycTests; 2001.

6. Tallberg IM, Ivachova E, Jones Tinghag K, Ostberg P. Swedish norms for word fluency tests: FAS, animals and verbs. Scand J Psychol. 2008;49(5):479-85.

7. Wechsler D. WAIS-IV. Manual del 1 – svensk version. Stockholm, Sweden 2011.

**Supplementary table 2
Results from cognitive testing, pre-operatively and one year post-operatively**

|  | |  |  | **PRE-OP** | | **POST-OP**  **One year** | |
| --- | --- | --- | --- | --- | --- | --- | --- |
| **COGNITIVE DOMAIN** | | **Test variable** | **n** | **Patients  mean (SD)** | **Patients median  (q1-q3)** | **Patients mean (SD)** | **Patients median (q1-q3)** |
| **LEARNING/ MEMORY** | Visuo-spatial | BVMT-R Learning | 126 | 22.8 (6.5) | 24 (18-28) | 23.2 (7.2) | 25 (17-29) |
|  |  | BVMT-R Del. recall | 126 | 9.1 (2.6) | 10 (8-11) | 9.0 (2.8) | 10 (7-11) |
|  | Verbal | RAVLT Learning | 126 | 51.9 (10.2) | 53 (45-60) | 50.3 (13.0) | 51 (42.75-60) |
|  |  | RAVLT Del. recall | 125 | 11.2 (3.2) | 12 (9-14) | 10.9 (3.7) | 11 (8-14) |
| **VISUOSPAT/ -PERCEPT.** | Speed | Trail Making Test A | 85 | 31.3 (12.1) | 30 (22.5-36) | 32.5 (12.7) | 31 (24.5-37) |
|  | Exec. | Rey CFT copy | 109 | 33.4 (4.4) | 35 (32.5-36) | 33.5 (4.1) | 35 (33-36) |
| **LANGUAGE** | Visuo- perc. | Boston Naming Test | 105 | 51.7 (7.0) | 54 (49-56) | 51.3 (7.5) | 54 (48.5-57) |
|  | Exec. | Phonemic fluency | 110 | 38.8 (14.8)┼ | 39 (27-49.25) | 38.1 (15.7)┼ | 37.5 (26-50.25) |
|  | Speed | Semantic fluency | 108 | 22.9 (7.4) | 23 (18.25-28) | 22.6 (9.4) | 22 (18-28) |
|  |  | CWIT 1 | 105 | 32.7 (9.0) | 30 (28-35) | 35.1 (14.9) | 31 (28-38) |
|  |  | CWIT 2 | 101 | 23.7 (5.0) | 22 (20-25.5) | 26.8 (14.6) | 24 (20.5-29) |
| **EXECUTIVE** | Language | CWIT 3 | 105 | 57.5 (21.4)┼ | 53 (45-62) | 64.7 (45.0) | 55 (45.5-68) |
|  |  | CWIT 4 | 105 | 67.5 (23.7) | 60 (53.5-72.5) | 78.5 (55.5) | 59 (51-82) |
|  | Visuo- spatial | Trail Making Test B | 85 | 83.3 (52.3) | 68 (64.5-89) | 83.6 (44.2) | 76 (56-96.5) |
|  | Attent | Digit span backward | 126 | 6.1 (1.1) | 6 (5-7) | 5.9 (1.2) | 6 (5-7) |
| **SPEED/ ATTENTION** | Attent. | Digit span forward | 126 | 4.5 (1.2) | 5 (4-5) | 4.5 (1.3) | 4 (3-5) |
|  | Speed | Coding | 86 | 59.7 (14.0)┼ | 59 (51.25-69) | 59.3 (15.7)┼ | 60 (50-70.25) |

Results presented per test variable. Abbreviations: BVMT-R = Brief Visuospatial Memory Test – Revised, CWIT = D-KEFS Color Word Interference Test, RAVLT = Rey Auditory Verbal Learning Test, Rey CFT = Rey Complex Figure Test copying. Data was not considered normally distributed, except for ┼.

**Supplementary table 3
Proportions of patients with significantly declined, improved or unchanged results**

| **COGNITIVE DOMAIN** | | **Test variable** | **N** | **CHANGE (%) ∆Z > 1** | | | **RCI (%)  > 1.645** | | |
| --- | --- | --- | --- | --- | --- | --- | --- | --- | --- |
|  |  |  |  | **Declined** | **Stable** | **Improved** | **Declined** | **Stable** | **Improved** |
| **LEARNING/ MEMORY** | Visuo-spatial | BVMT-R Learning | 126 | 16.7 | 58.7 | 24.6 | 11.9 | 73.8 | 14.3 |
|  |  | BVMT-R Del. recall | 126 | 22.2 | 54.8 | 23.0 | 18.3 | 69.8 | 11.9 |
|  | Verbal | RAVLT Learning | 125 | 23.8 | 59.5 | 16.7 | 26.4 | 65.6 | 8.0 |
|  |  | RAVLT Del. recall | 125 | 20.8 | 64.8 | 14.4 | 28.0 | 67.2 | 4.8 |
| **VISUOSPAT/ -PERCEPT.** | Speed | Trail Making Test A | 85 | 12.9 | 73.0 | 14.1 | 18.8 | 74.1 | 7.1 |
|  | Exec. | Rey CFT copy | 109 | N.A. | N.A. | N.A. | N.A. | N.A. | N.A. |
| **LANGUAGE** | Visuo- perc. | Boston Naming Test | 105 | 18.1 | 54.3 | 27.6 | 27.6 | 64.8 | 7.6 |
|  | Exec. | Phonemic fluency | 110 | 29.1 | 47.3 | 23.6 | 21.8 | 70.9 | 7.3 |
|  | Speed | Semantic fluency | 109 | 25.0 | 57.4 | 17.6 | 16.5 | 76.1 | 7.3 |
|  |  | CWIT 1 | 105 | 18.1 | 67.6 | 14.3 | 28.6 | 63.8 | 7.6 |
|  |  | CWIT 2 | 101 | 15.8 | 79.2 | 5.0 | 18.8 | 78.2 | 3.0 |
| **EXECUTIVE** | Language | CWIT 3 | 105 | 16.2 | 71.4 | 12.4 | 17.1 | 80.0 | 2.9 |
|  |  | CWIT 4 | 105 | 21.0 | 65.7 | 13.3 | 32.4 | 60.0 | 7.6 |
|  | Visuo- spatial | Trail Making Test B | 85 | 24.7 | 57.7 | 17.6 | 22.4 | 64.7 | 12.9 |
|  | Attent | Digit span backward | 126 | 15.9 | 70.6 | 13.5 | 13.5 | 84.9 | 1.6 |
| **SPEED/ ATTENTION** | Attent | Digit span forward | 126 | 15.9 | 75.4 | 8.7 | 12.7 | 87.3 | 0 |
|  | Speed | Coding | 86 | 15.1 | 64.0 | 20.9 | 18.6 | 68.6 | 12.8 |

Data presented per domain and sub-domain. Abbreviations: BVMT-R = Brief Visuospatial Memory Test – Revised, CWIT = D-KEFS Color Word Interference Test, RAVLT = Rey Auditory Verbal Learning Test, Rey CFT = Rey Complex Figure Test copying.

**Supplementary table 4.
Logistic regression models for identifying predictors of cognitive decline per domain.**

|  | **Covariate** | **Univariable OR (95% CI)** | ***P*-value** | **Multivariable,  OR (95% CI)** | ***P*-value** |
| --- | --- | --- | --- | --- | --- |
| **LEARNING/ MEMORY** | Age | 1.06 (1.01-1.10) | 0.008* | 1.07 (1.02-1.12) | 0.007* |
|  | KPS pre-operative | 1.02 (0.97-1.07) | 0.561 | 1.03 (0.96-1.09) | 0.416 |
|  | Tumor size | 1.00 (0.99-1.01) | 0.380 | 1.00 (0.99-1.01) | 0.616 |
|  | 1p19q codeletion | 0.70 (0.30-1.67) | 0.422 | 0.55 (0.20-1.54) | 0.257 |
|  | High grade | 0.66 (0.25-1.70) | 0.385 | 1.09 (0.35-3.35) | 0.887 |
|  | HADS >8 p preop | 1.22 (0.47-3.19) | 0.680 | 1.30 (0.47-3.62) | 0.616 |
|  | Chemoradiotherapy | 1.31 (0.45-3.85) | 0.624 | 1.34 (0.38-4.76) | 0.649 |
| **VISUO-/**  **PERCEPTUAL** | Age | 1.02 (0.98-1.07) | 0.349 | 1.03 (0.98-1.10) | 0.252 |
|  | KPS pre-operative | 0.97 (0.91-1.04) | 0.407 | 0.93 (0.84-1.03) | 0.170 |
|  | Tumor size | 0.99 (0.98-1.01) | 0.233 | 0.99 (0.97-1.01) | 0.150 |
|  | 1p19q codeletion | 0.69 (0.23-1.12) | 0.521 | 0.37 (0.94-1.46) | 0.156 |
|  | High grade | 0.85 (0.27-2.74) | 0.788 | 0.89 (0.22-3.57) | 0.865 |
|  | HADS >8 p preop | 1.96 (0.57-6.74) | 0.285 | 1.92 (0.48-7.72) | 0.360 |
|  | Chemoradiotherapy | 0.51 (0.15-1.73) | 0.279 | 0.75 (0.16-3.47) | 0.709 |
| **LANGUAGE** | Age | 1.04 (1.00.1.08) | 0.044* | 1.04 (1.00-1.08) | 0.077 |
|  | KPS pre-operative | 1.00 (0.96-1.04) | 1.000 | 1.02 (0.96-1.07) | 0.571 |
|  | Tumor size | 1.00 (0.99-1.01) | 0.758 | 1.00 (0.99-1.01) | 0.944 |
|  | 1p19q codeletion | 1.32 (0.61-2.84) | 0.483 | 1.22 (0.51-3.00) | 0.655 |
|  | High grade | 0.83 (0.38-1.82) | 0.637 | 0.82 (0.33-2.07) | 0.676 |
|  | HADS >8 p preop | 1.81 (0.75-4.38) | 0.187 | 1.77 (0.69-1.51) | 0.234 |
|  | Chemoradiotherapy | 2.86 (1.01-8.12) | 0.049* | 3.56 (1.08-11.71 | 0.037* |
| **EXECUTIVE** | Age | 1.05 (1.01-1.09) | 0.006* | 1.06 (1.01-1.11) | 0.013* |
|  | KPS pre-operative | 0.97 (0.93-1.01) | 0.168 | 1.00 (0.94-1.05) | 0.863 |
|  | Tumor size | 1.00 (0.99-1.01) | 0.818 | 0.99 (0.98-1.01) | 0.295 |
|  | 1p19q codeletion | 1.78 (0.82-3.81) | 0.146 | 1.71 (0.69-4.26) | 0.245 |
|  | High grade | 1.47 (0.65-3.32) | 0.352 | 1.65 (0.63-4.36) | 0.311 |
|  | HADS >8 p preop | 1.18 (0.47-2.93) | 0.727 | 1.03 (0.37-2.83) | 0.960 |
|  | Chemoradiotherapy | 2.60 (0.89-7.59) | 0.080 | 4.34 (1.12-16-78) | 0.033* |
| **SPEED/**  **ATTENTION** | Age | 1.02 (0.97-1.06) | 0.475 | 1.03 (0.98-1.09) | 0.278 |
|  | KPS pre-operative | 1.06 (0.99-1.13) | 0.096 | 1.08 (1.00-1.18) | 0.066 |
|  | Tumor size | 1.00 (0.99-1.01) | 0.977 | 0.99 (0.98-1.01) | 0.378 |
|  | 1p19q codeletion | 0.73 (0.27-1.98) | 0.536 | 0.50 (0.14-1.75) | 0.279 |
|  | High grade | 1.25 (0.45-3.41) | 0.670 | 2.07 (0.55-7.86) | 0.283 |
|  | HADS >8 p preop | 0.81 (0.24-2.70) | 0.725 | 0.71 (0.19-2.61) | 0.608 |
|  | Chemoradiotherapy | 1.57 (0.42-5.83) | 0.500 | 2.38 (0.42-13.38) | 0.324 |

Abbreviations: Age (per year), preoperative KPS (0-100), tumor size (per ml), presence of 1p19q-codeletion (no/yes), high grade (no/yes defined as WHO grade 2 vs 3-4), HADS score >8 p in either subscale preoperatively (no/yes), chemoradiotherapy meaning combined treatment with chemo- and radiotherapy (no/yes). OR = Odds ratios (95% CI). Significant p-values p < .05 are marked with *.
