# Supplementary figures and images for: Reliable cognitive changes the first year following guideline-based treatment of isocitrate dehydrogenase mutated gliomas: A longitudinal multicenter study
Source: Neuro Oncol. 2025 Nov 9;28(3):704–16. doi: 10.1093/neuonc/noaf263 (PMC13070499; doi:10.1093/neuonc/noaf263)

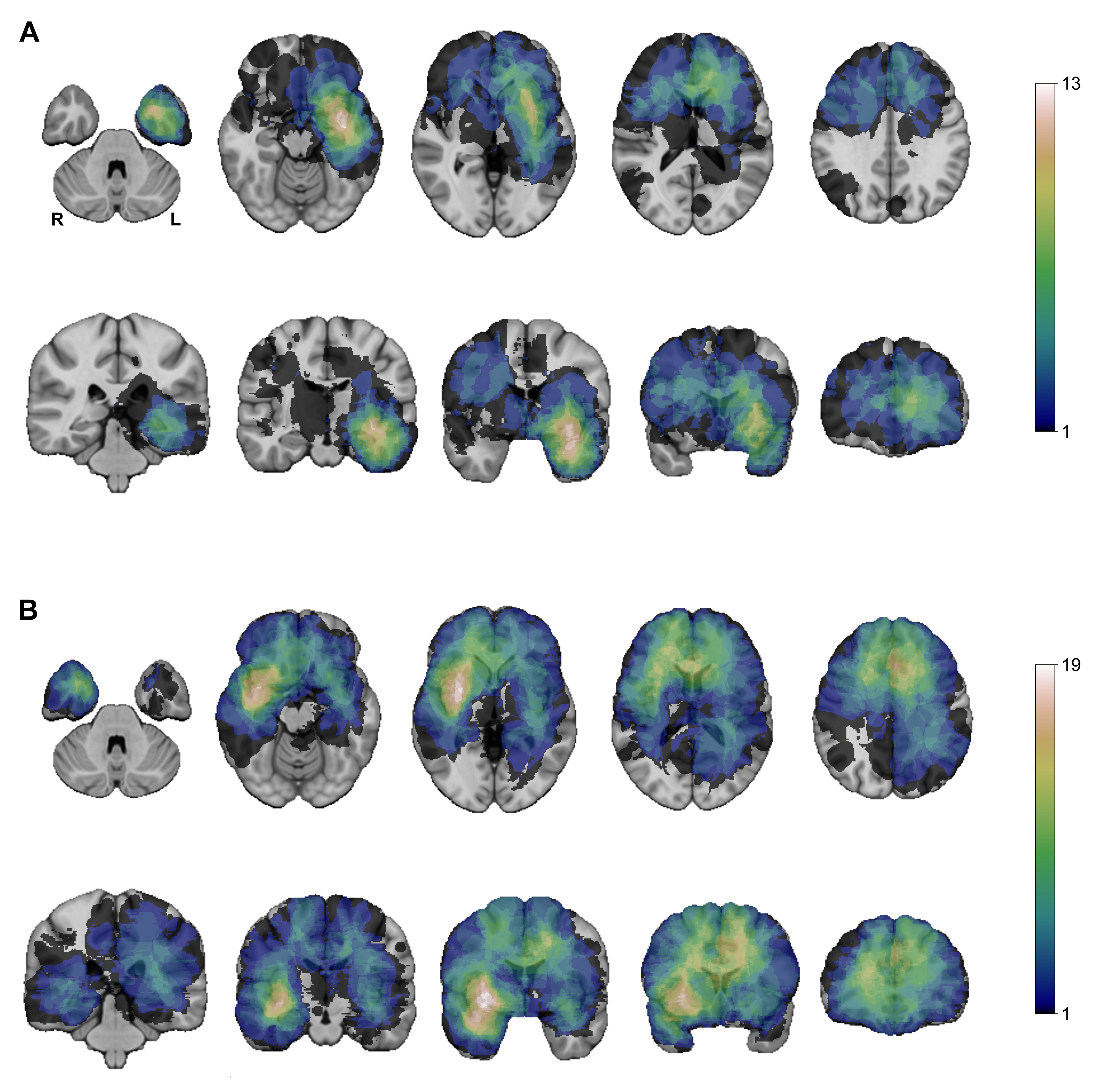

Supplement: noaf263_Supplementary_Data [file noaf263_supplementary_data.zip › SupplementaryFigure4.tiff]

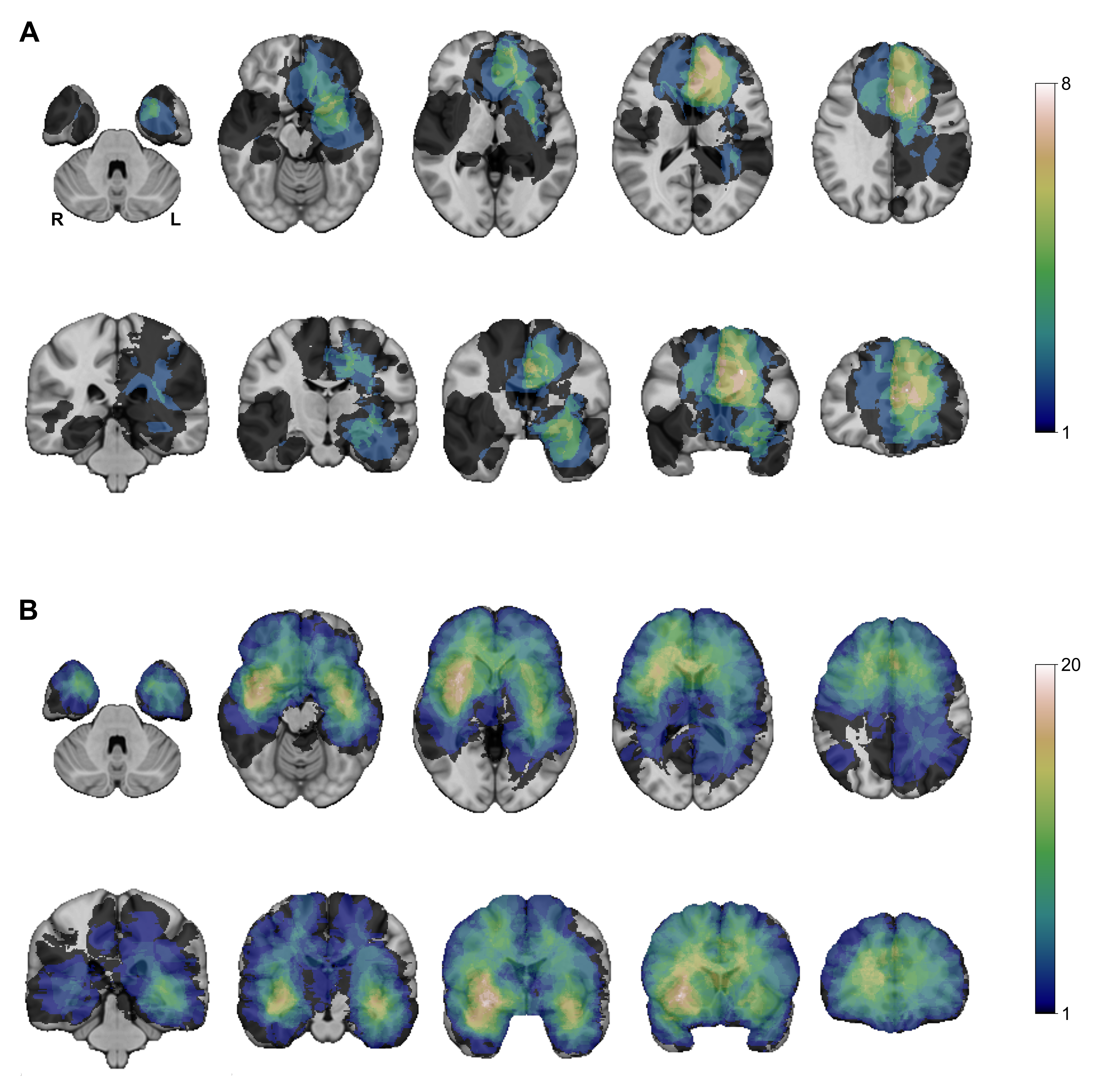

Supplement: noaf263_Supplementary_Data [file noaf263_supplementary_data.zip › SupplementaryFigure5.tiff]

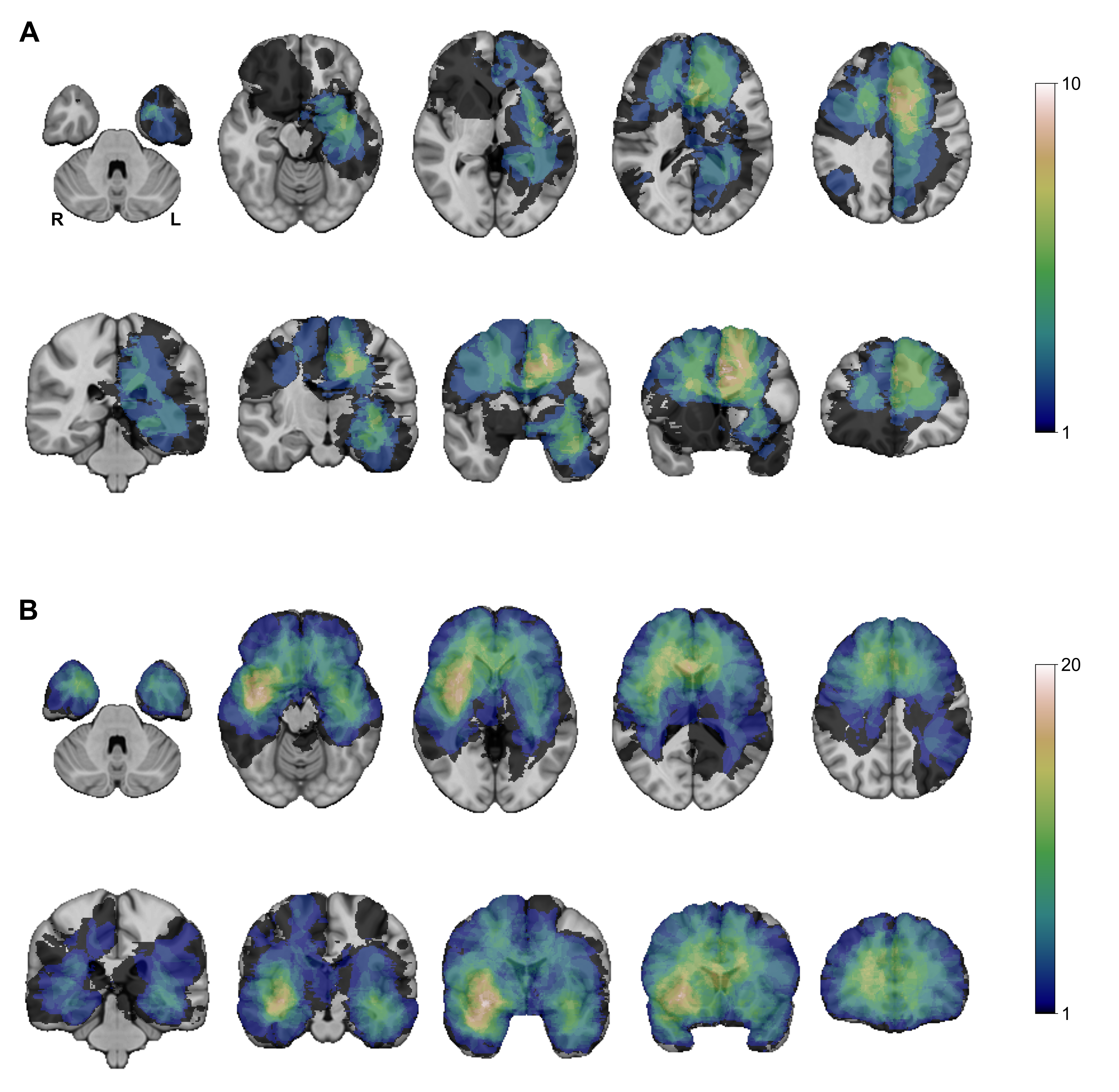

Supplement: noaf263_Supplementary_Data [file noaf263_supplementary_data.zip › SupplementaryFigure6.tiff]

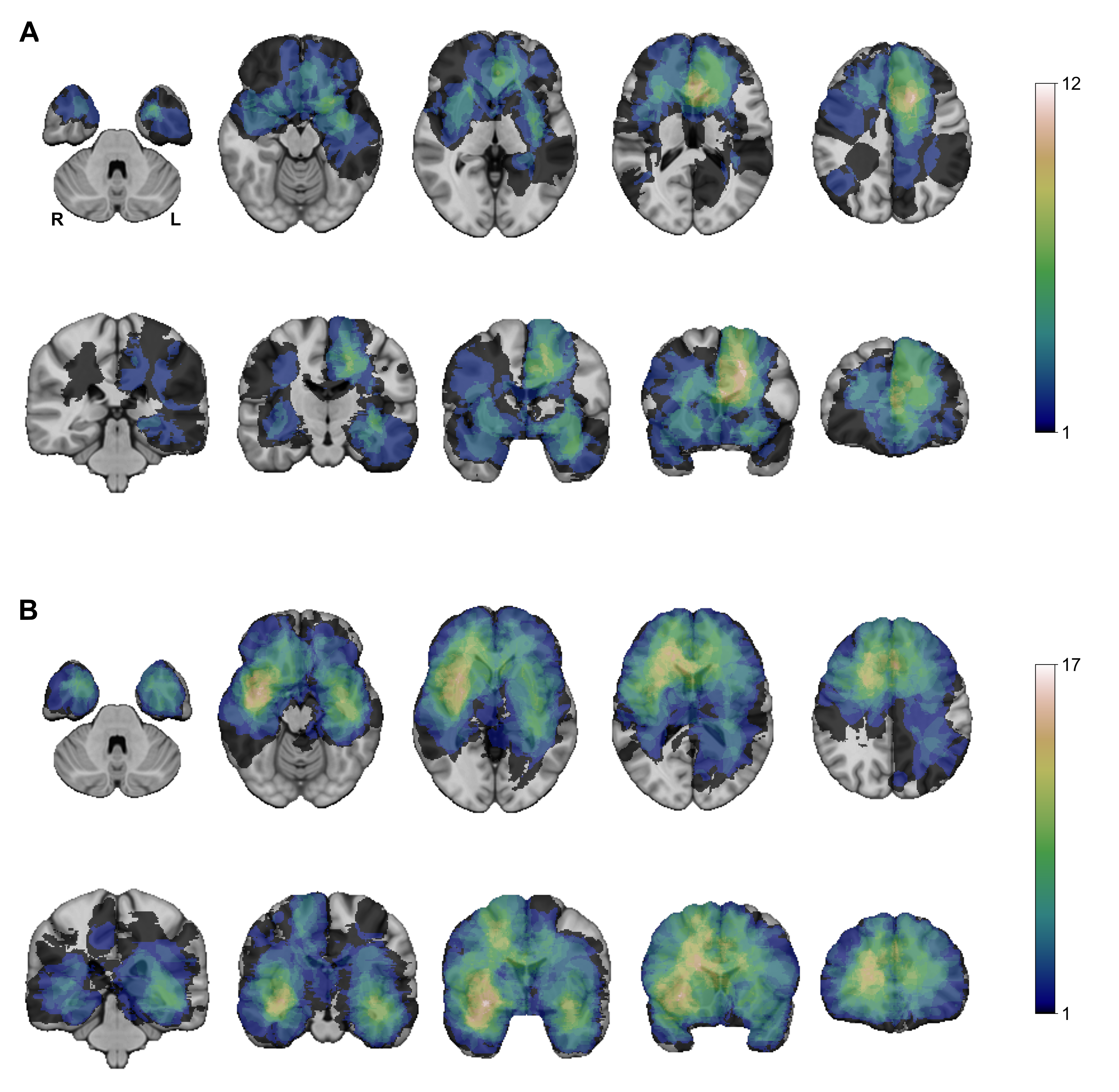

Supplement: noaf263_Supplementary_Data [file noaf263_supplementary_data.zip › SupplementaryFigure7.tiff]

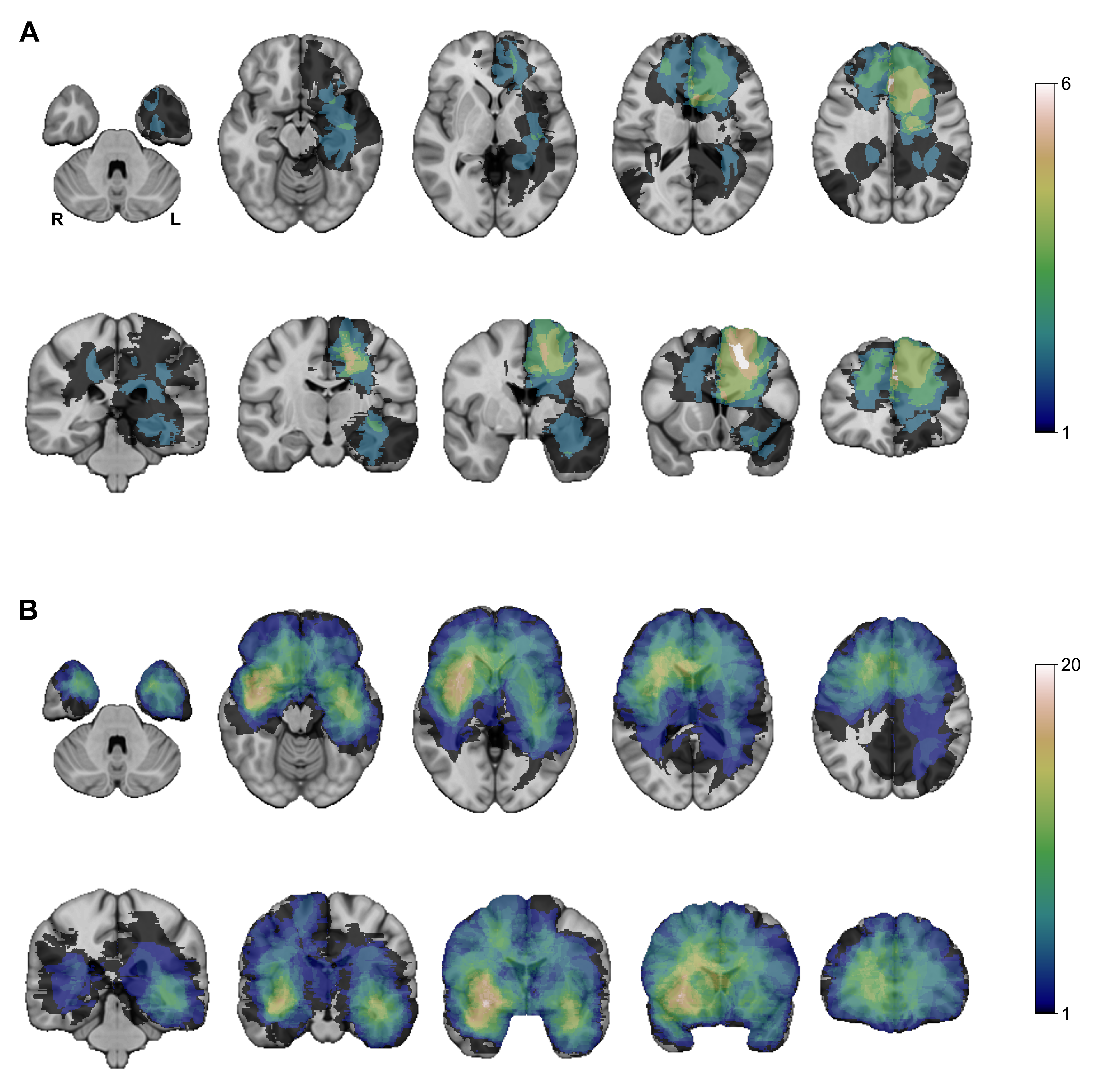

Supplement: noaf263_Supplementary_Data [file noaf263_supplementary_data.zip › SupplementaryFigure8.tiff]

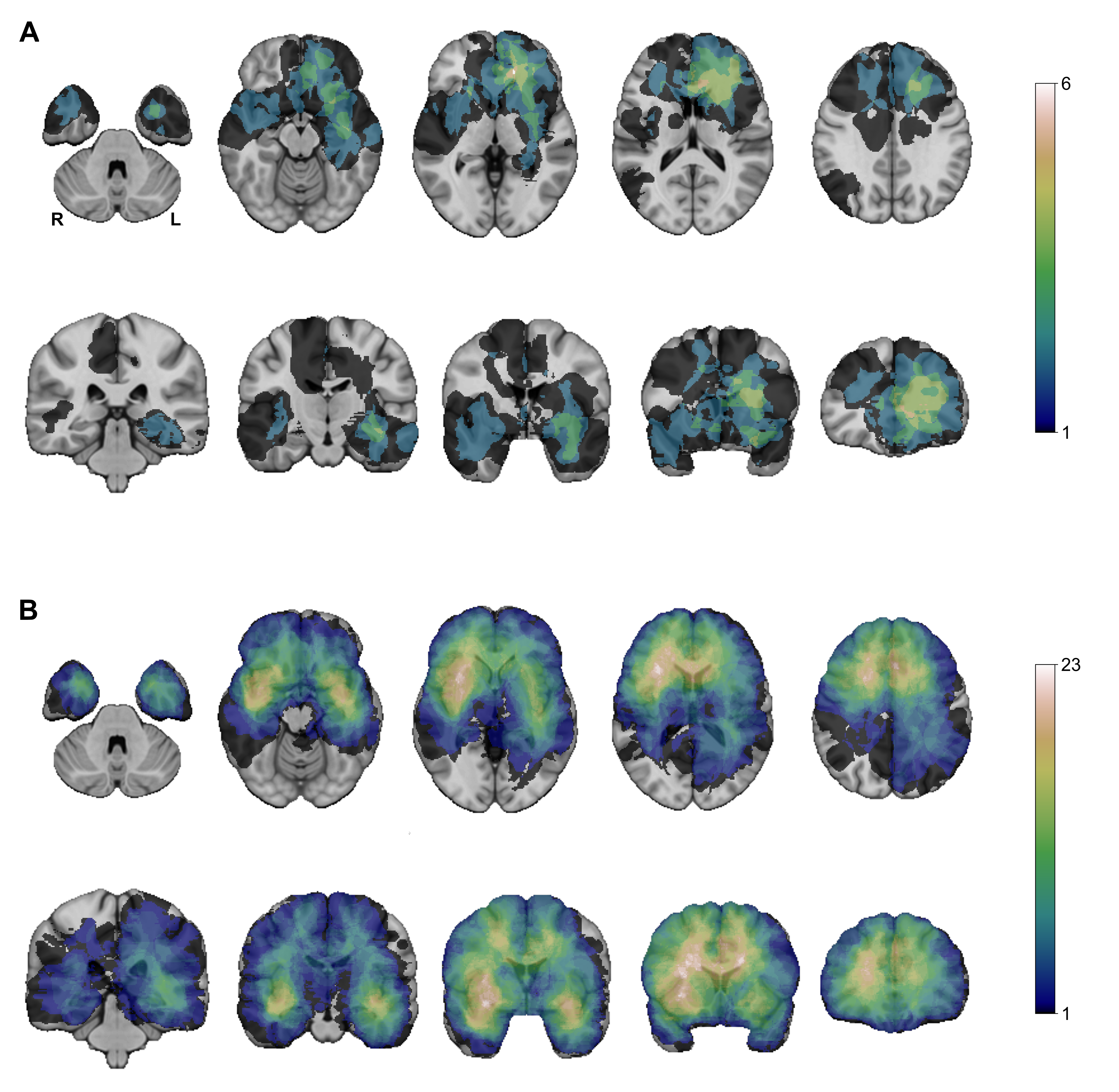

Supplement: noaf263_Supplementary_Data [file noaf263_supplementary_data.zip › SupplementaryFigure9.tiff]

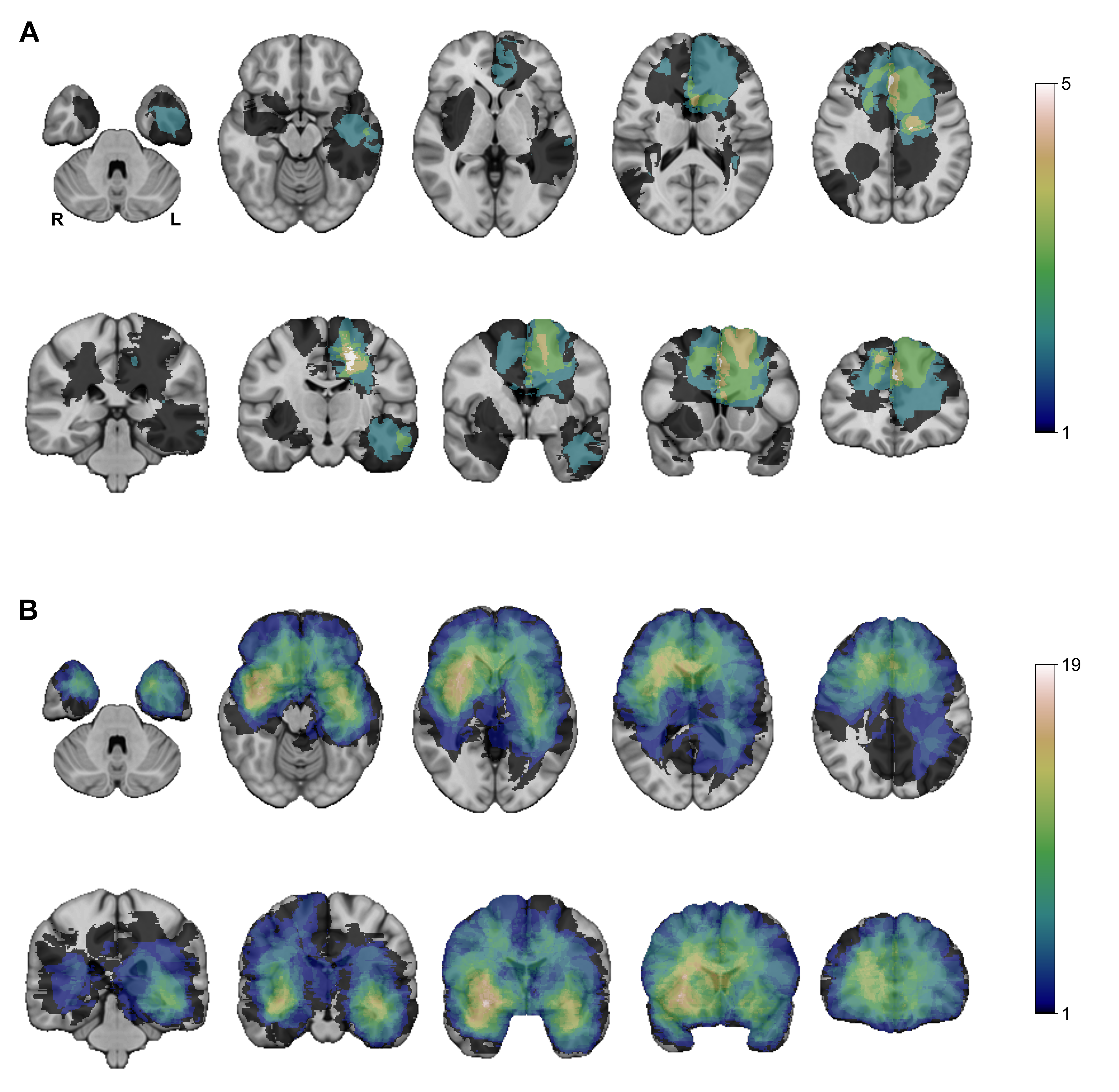

Supplement: noaf263_Supplementary_Data [file noaf263_supplementary_data.zip › SupplementaryFigure10.tiff]

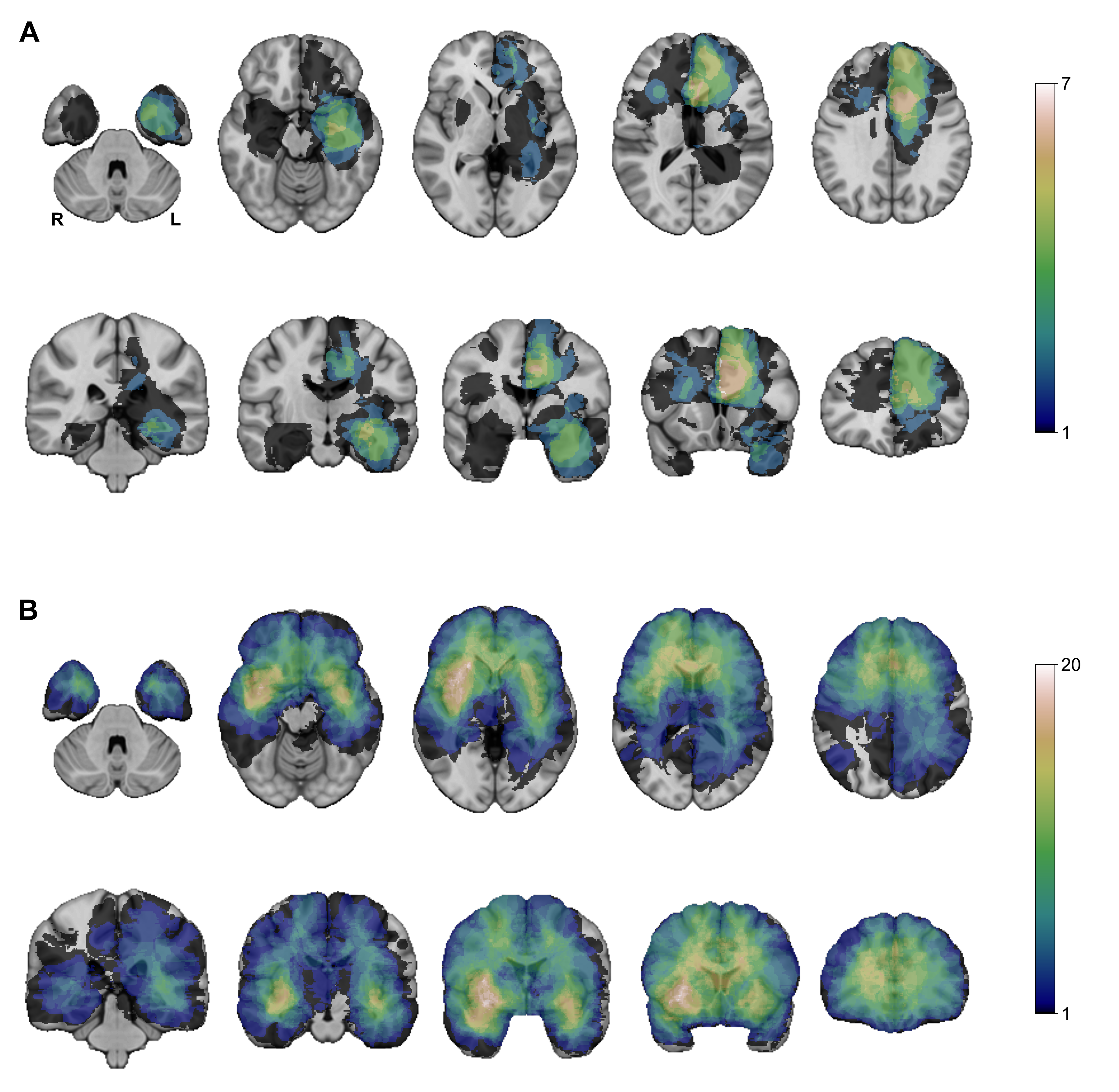

Supplement: noaf263_Supplementary_Data [file noaf263_supplementary_data.zip › SupplementaryFigure11.tiff]

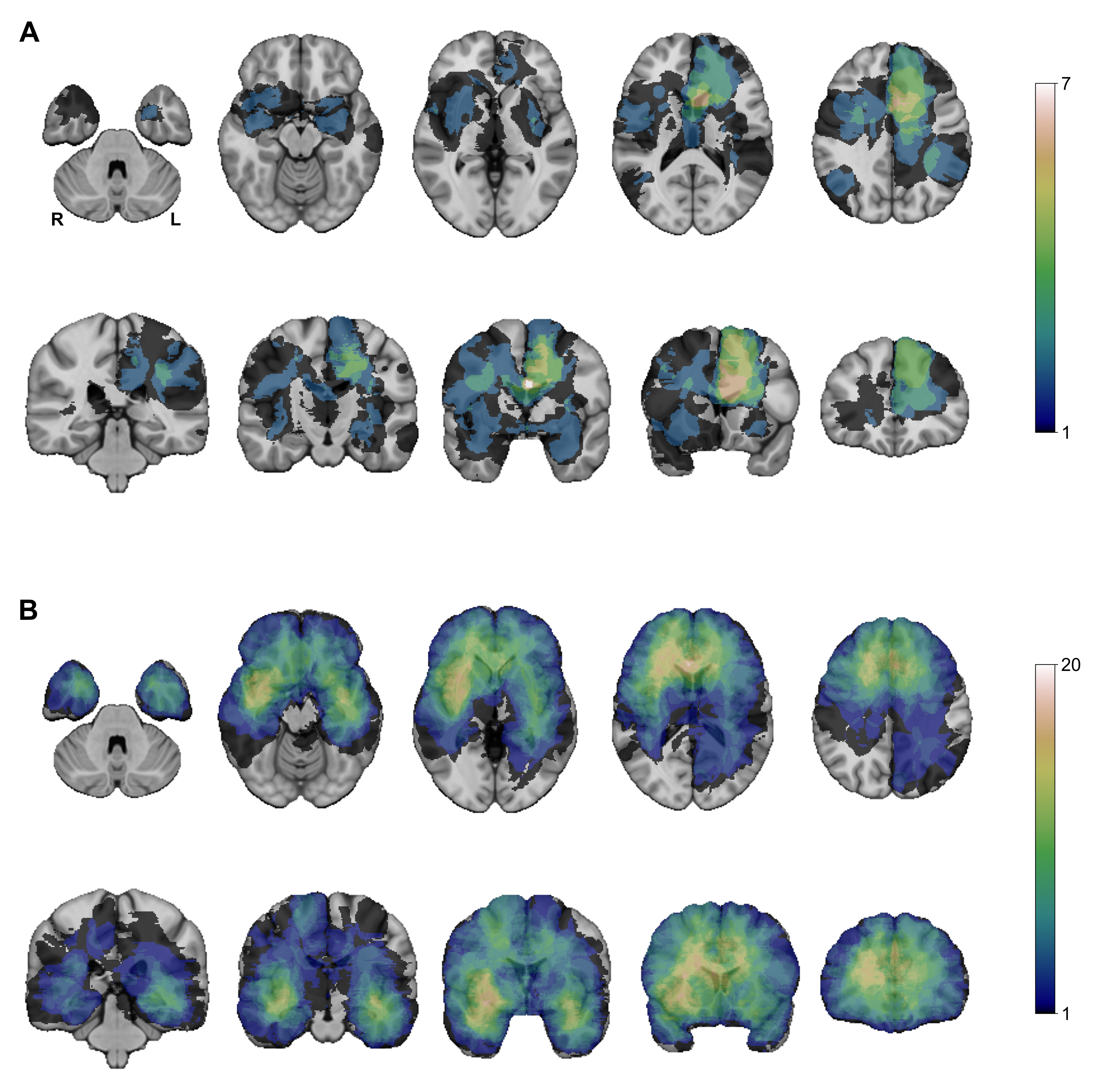

Supplement: noaf263_Supplementary_Data [file noaf263_supplementary_data.zip › SupplementaryFigure12.tiff]

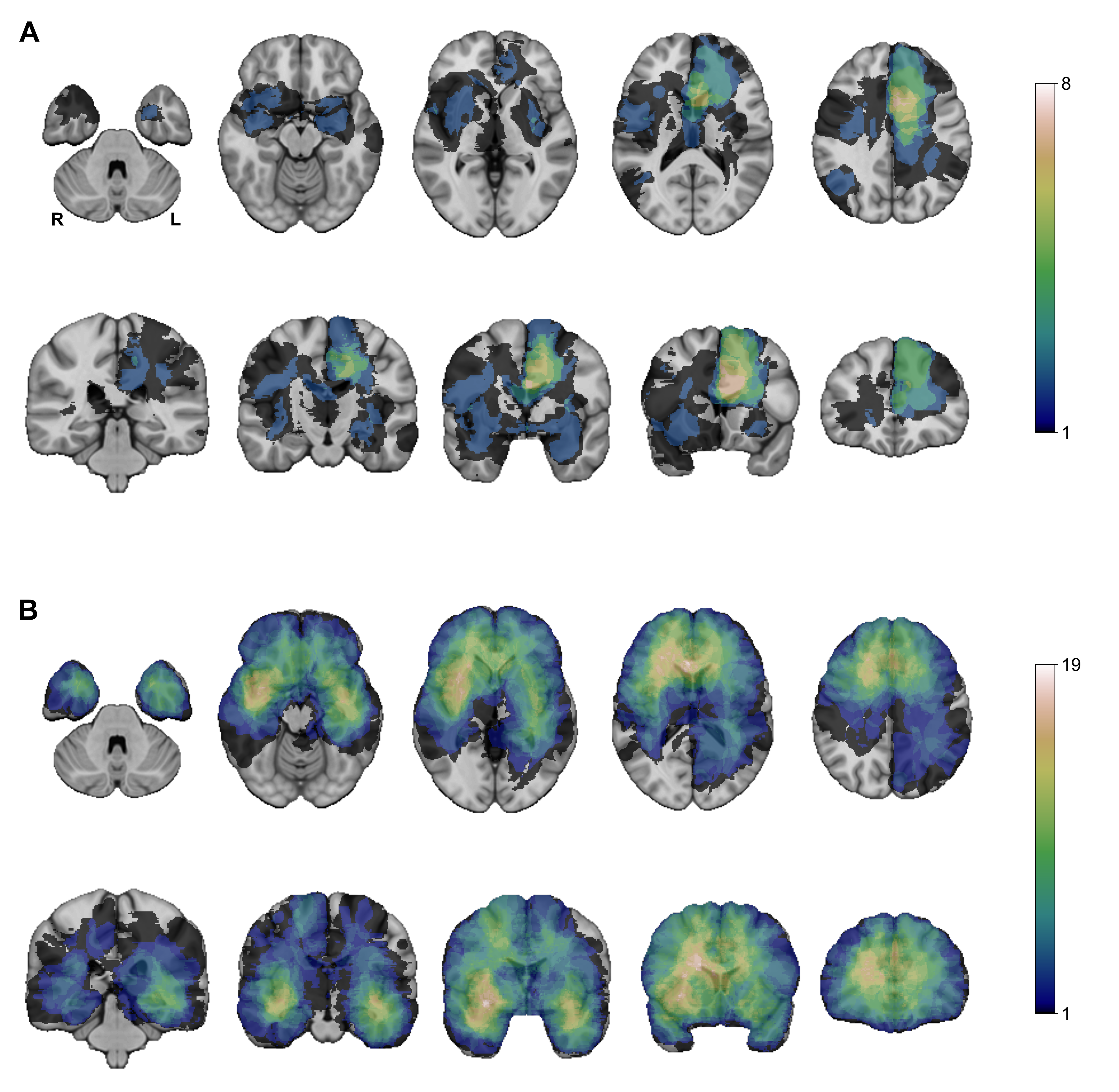

Supplement: noaf263_Supplementary_Data [file noaf263_supplementary_data.zip › SupplementaryFigure13.tiff]

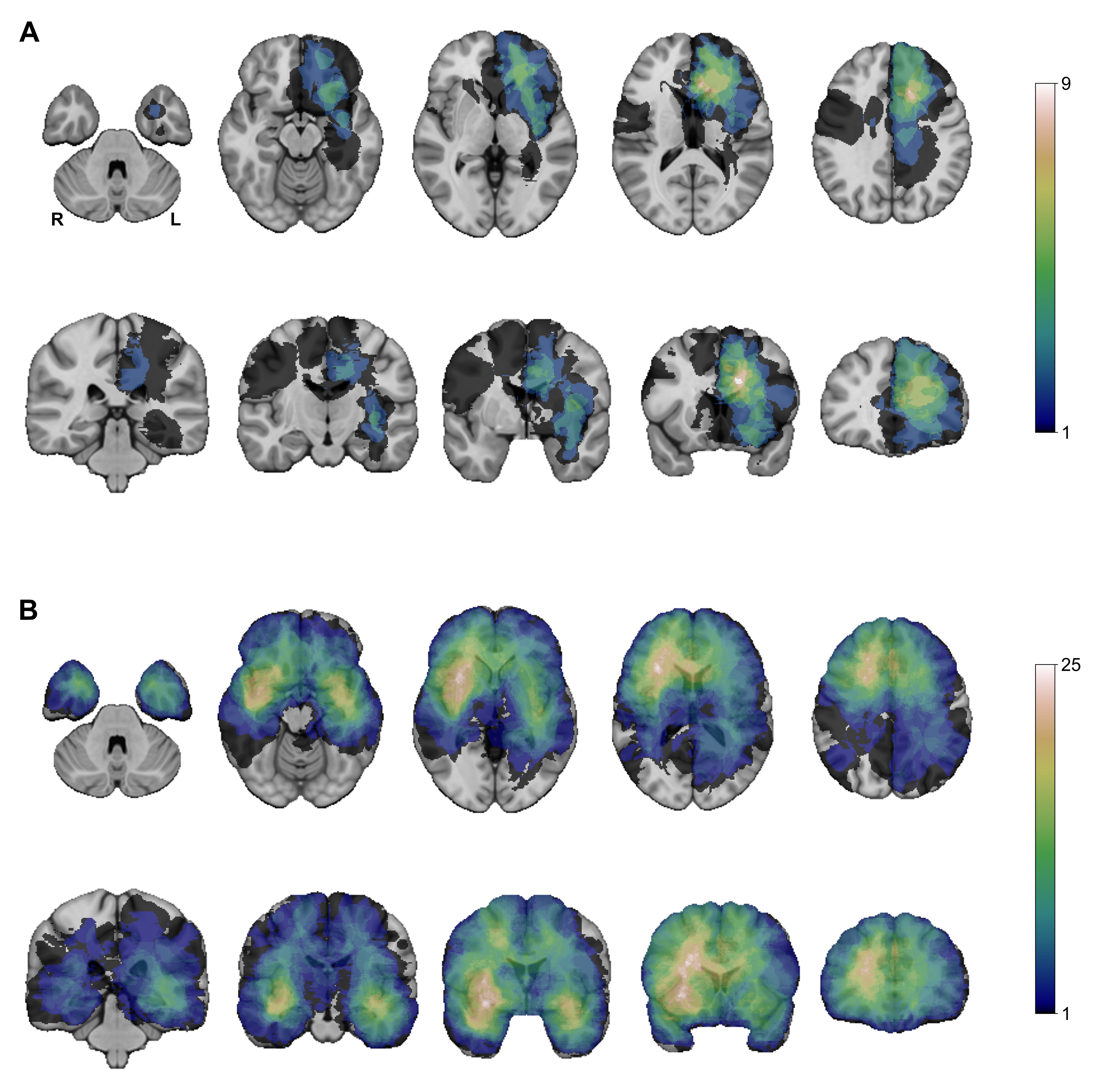

Supplement: noaf263_Supplementary_Data [file noaf263_supplementary_data.zip › SupplementaryFigure14.tiff]

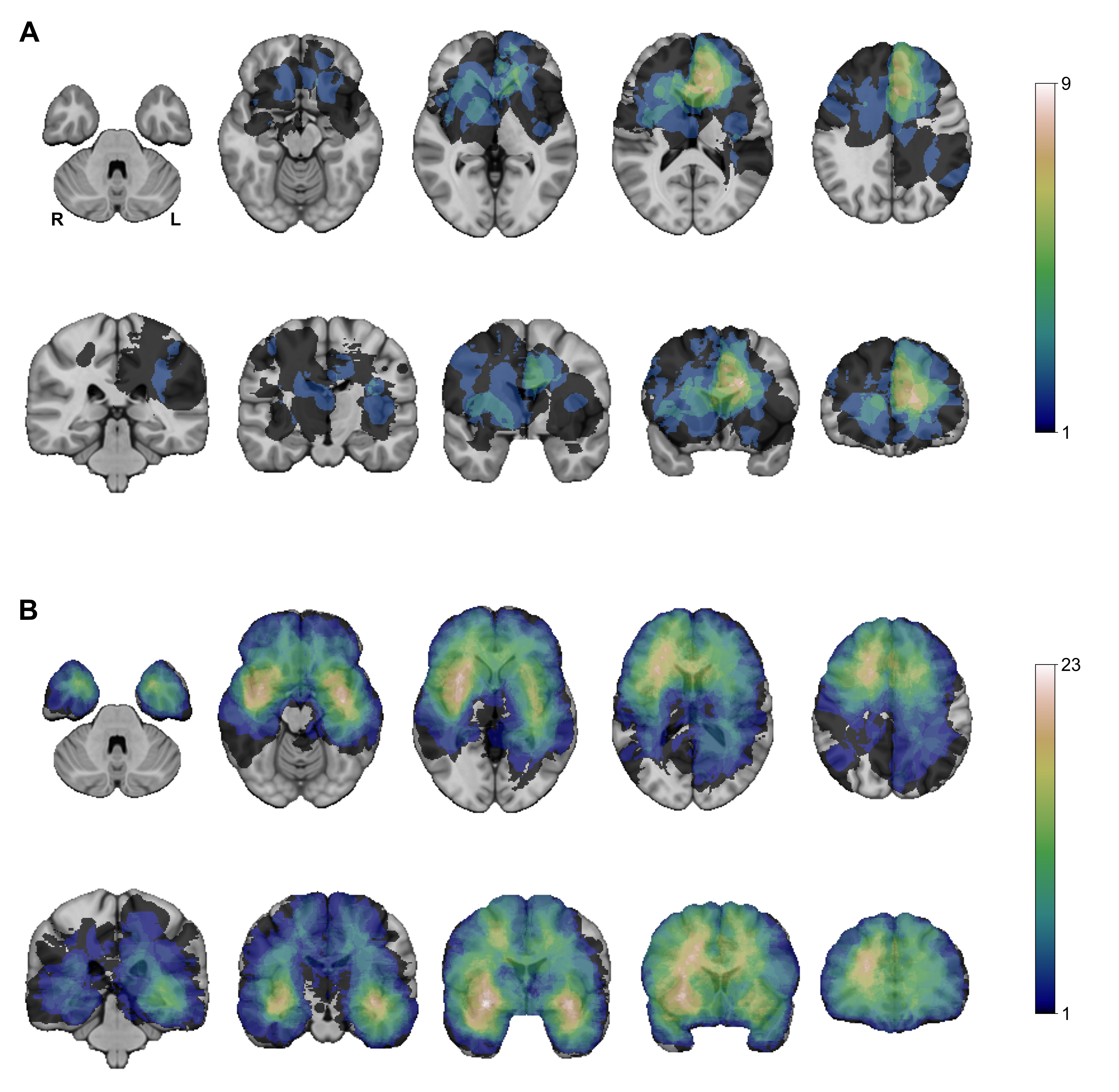

Supplement: noaf263_Supplementary_Data [file noaf263_supplementary_data.zip › SupplementaryFigure15.tiff]

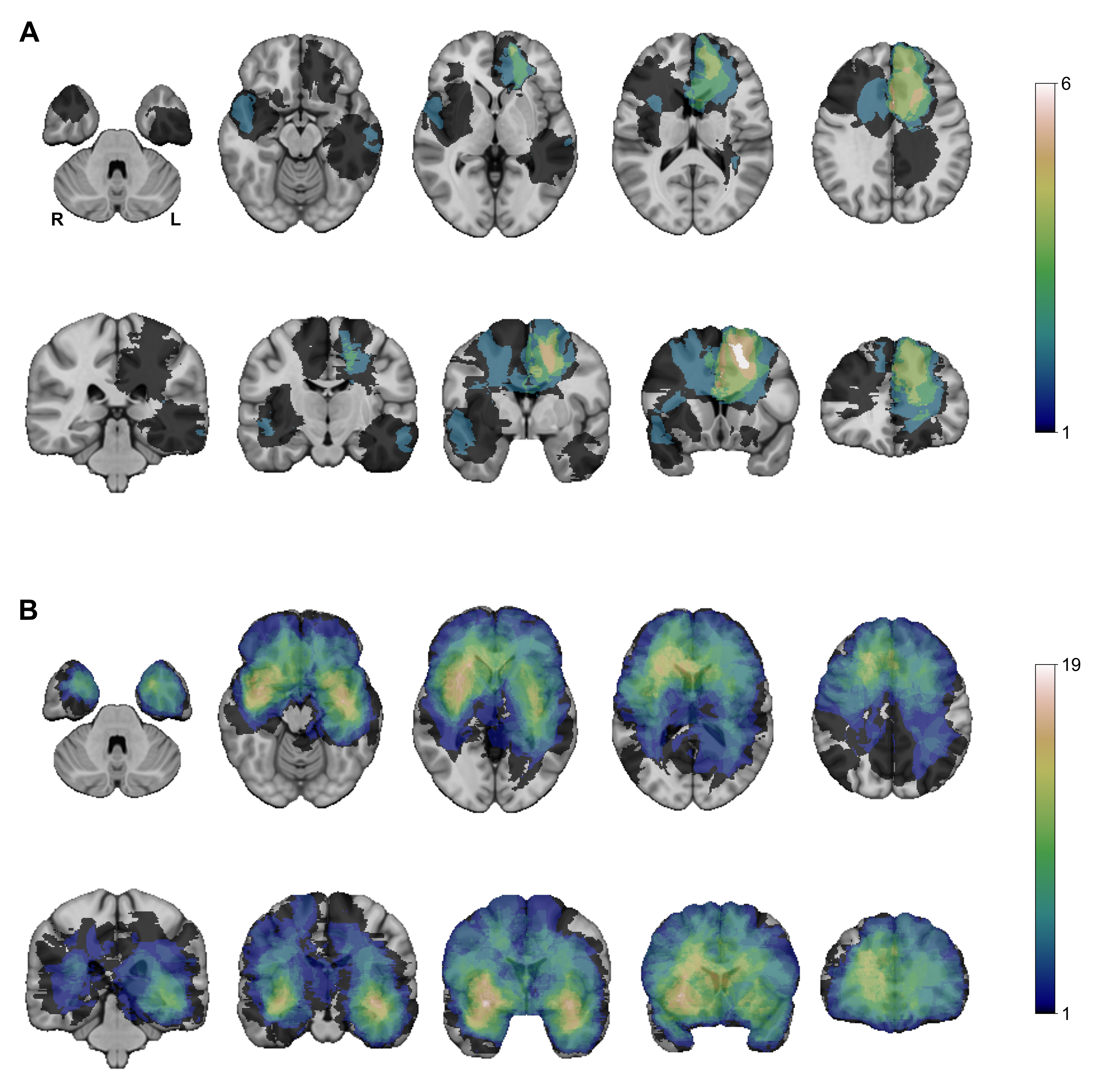

Supplement: noaf263_Supplementary_Data [file noaf263_supplementary_data.zip › SupplementaryFigure16.tiff]

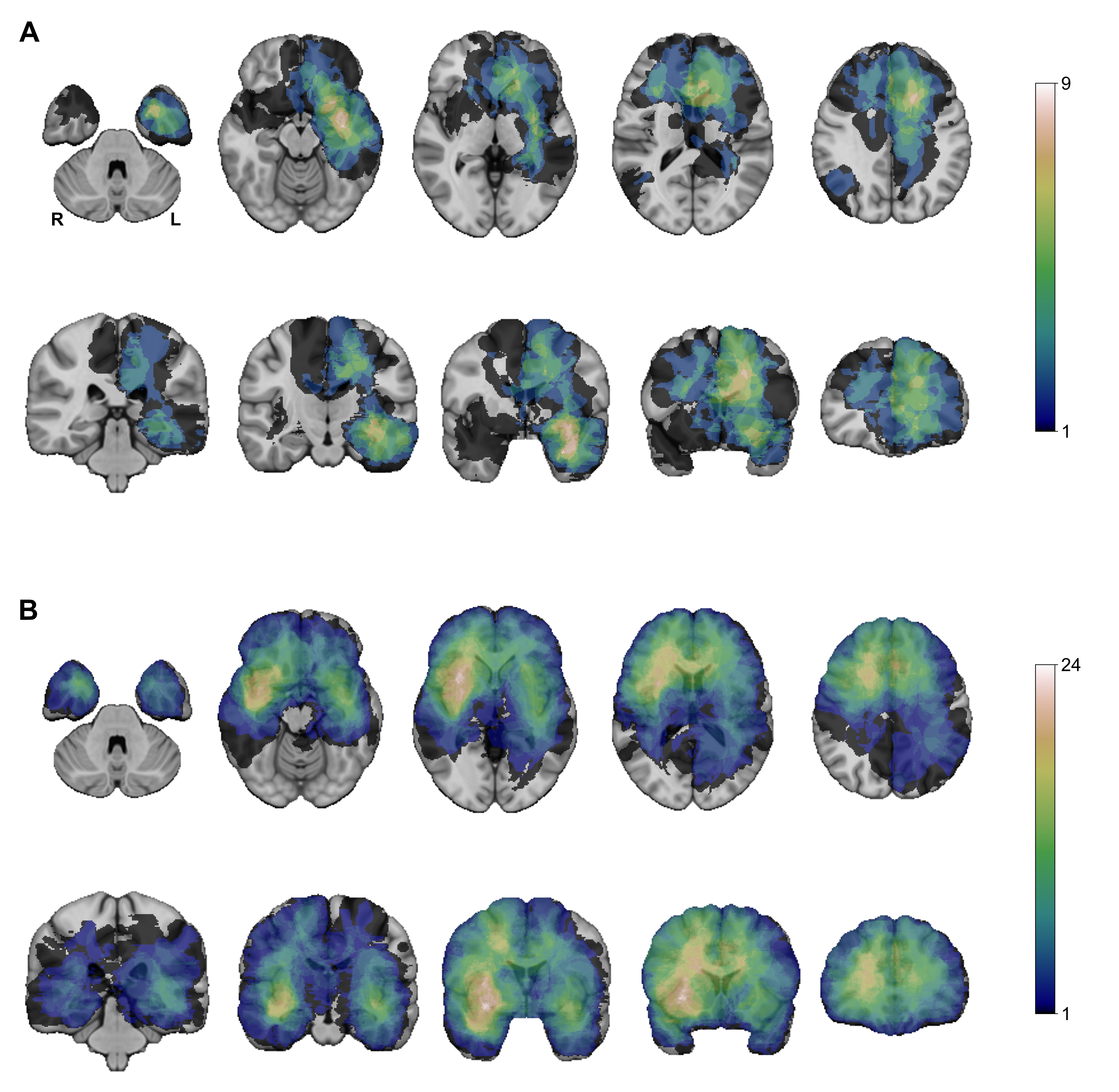

Supplement: noaf263_Supplementary_Data [file noaf263_supplementary_data.zip › SupplementaryFigure17.tiff]

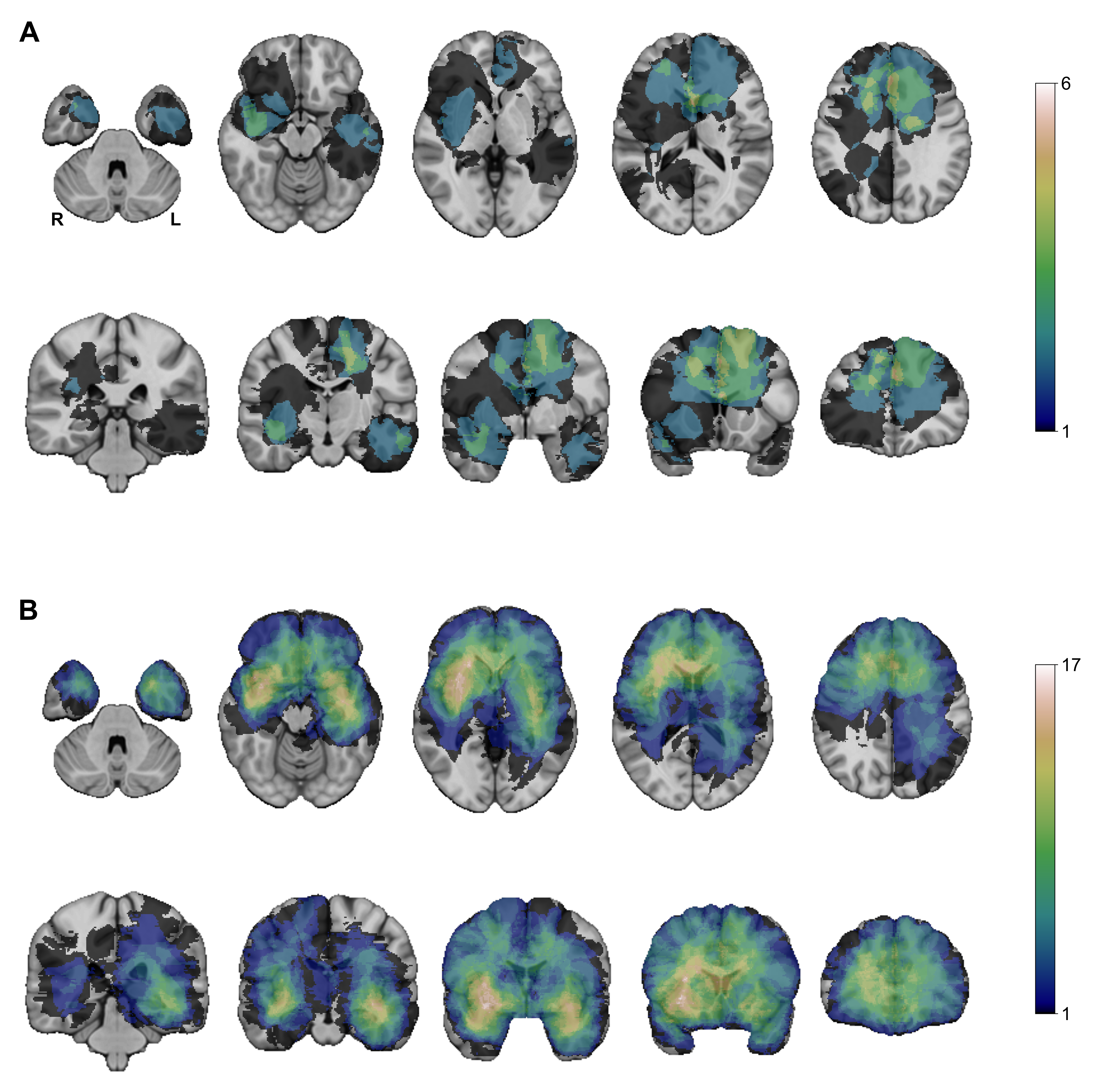

Supplement: noaf263_Supplementary_Data [file noaf263_supplementary_data.zip › SupplementaryFigure18.tiff]

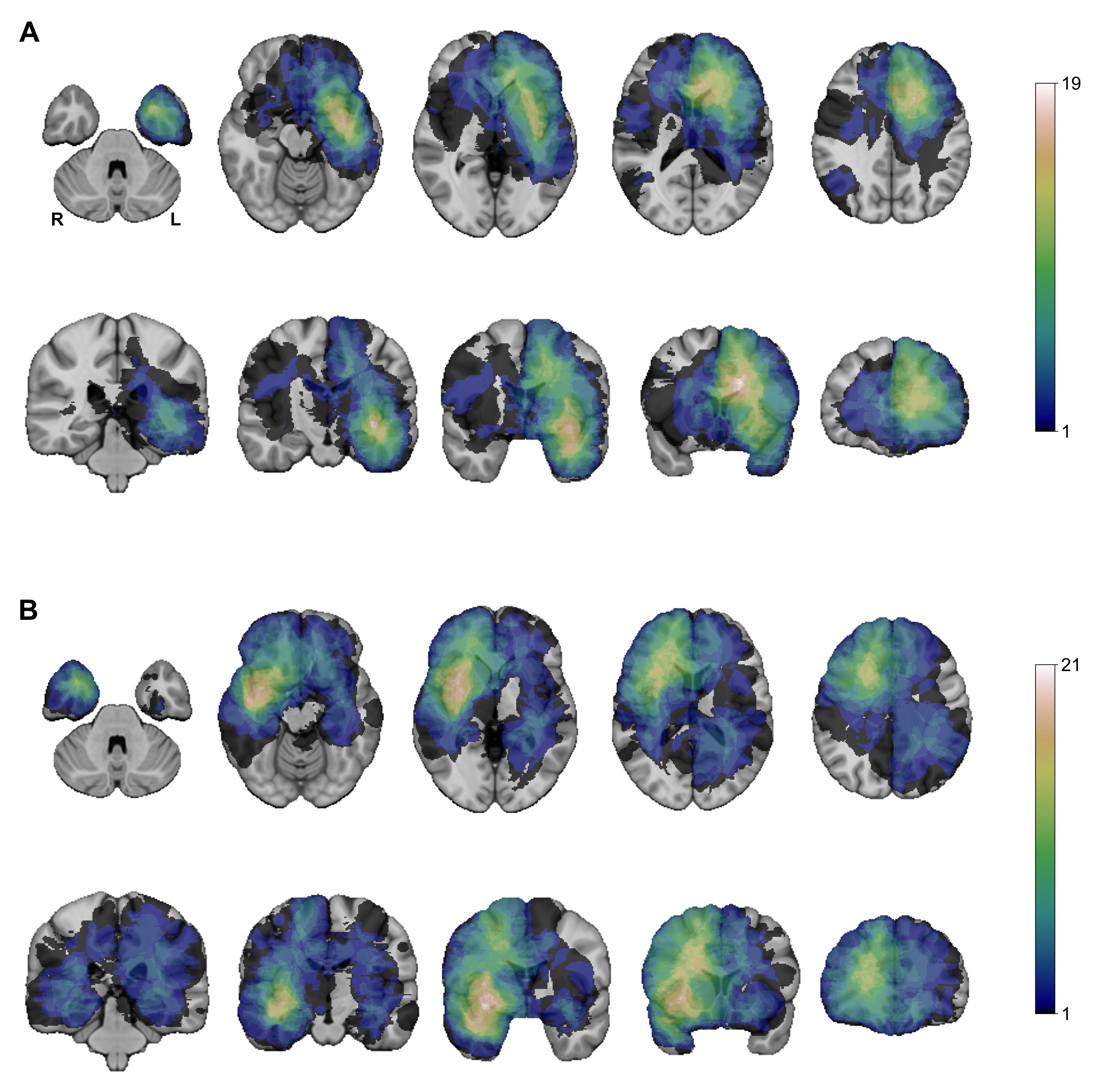

Supplement: noaf263_Supplementary_Data [file noaf263_supplementary_data.zip › SupplementaryFigure19.tiff]

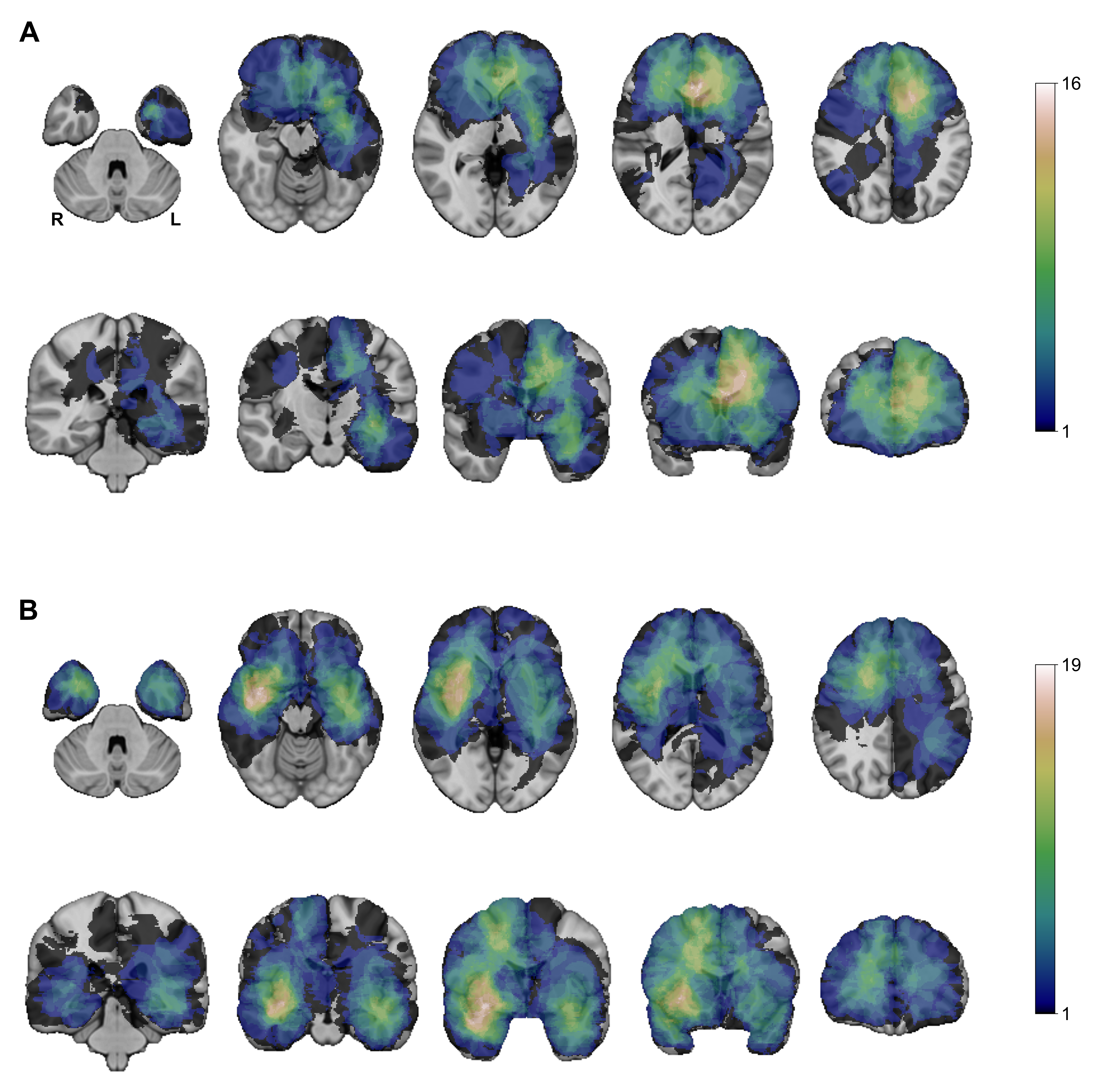

Supplement: noaf263_Supplementary_Data [file noaf263_supplementary_data.zip › SupplementaryFigure20.tiff]

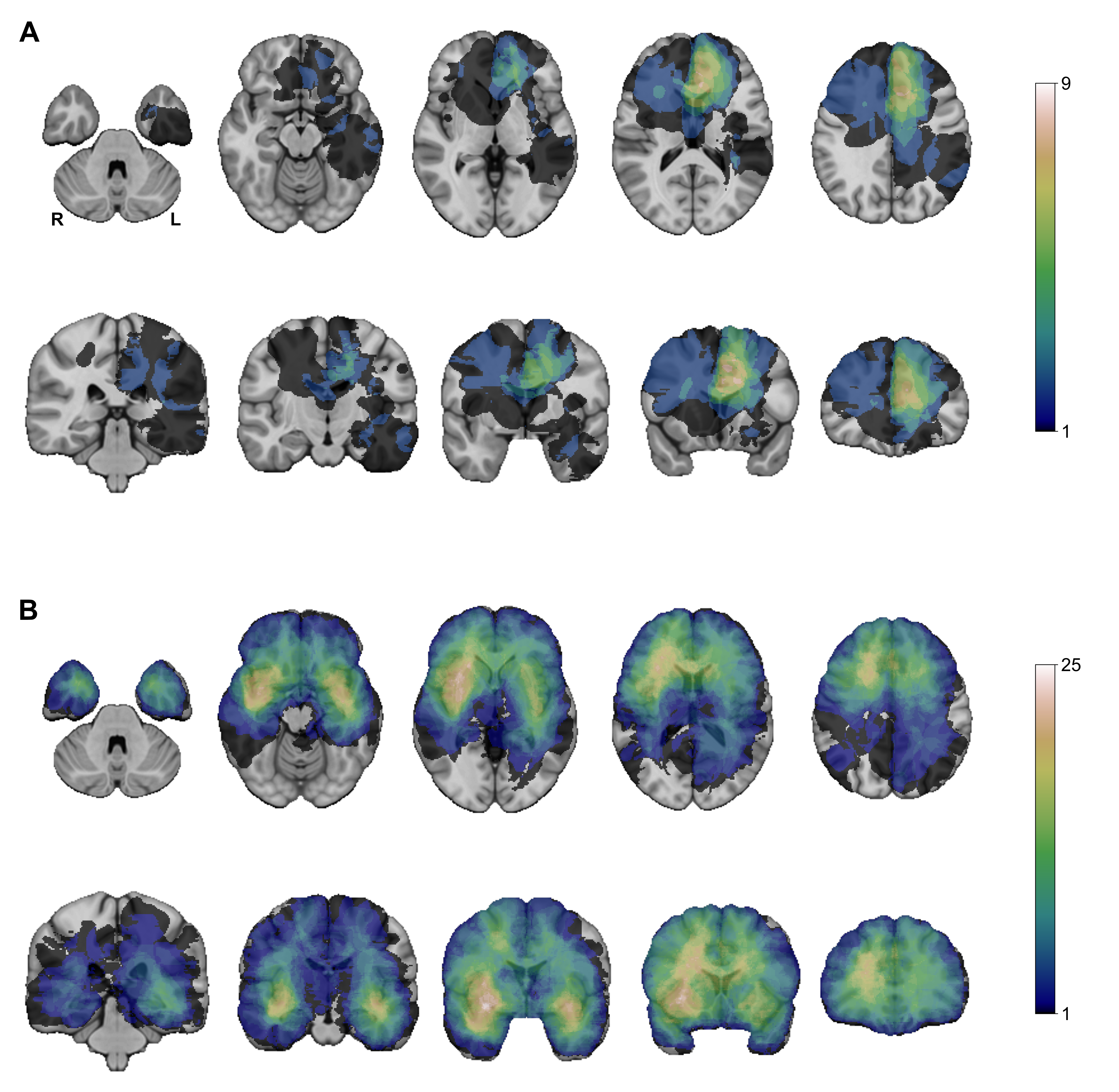

Supplement: noaf263_Supplementary_Data [file noaf263_supplementary_data.zip › SupplementaryFigure21.tiff]

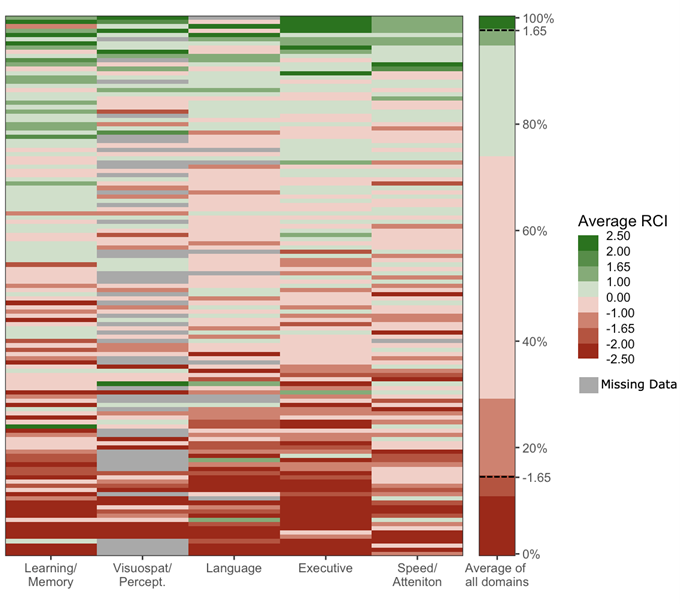

Supplement: noaf263_Supplementary_Data [file noaf263_supplementary_data.zip › SupplementaryFigure1.tif]

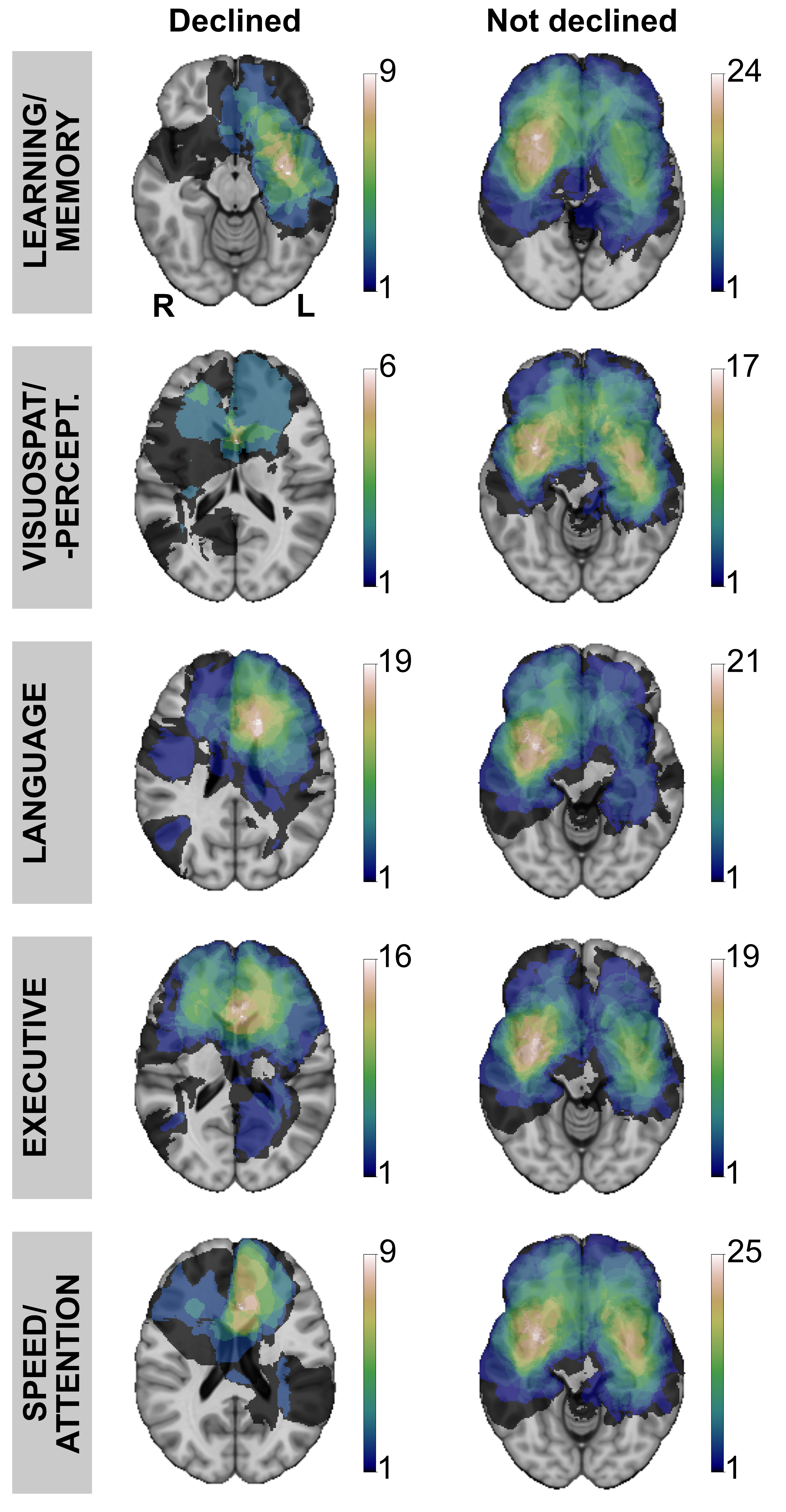

Supplement: noaf263_Supplementary_Data [file noaf263_supplementary_data.zip › SupplementaryFigure2.tiff]

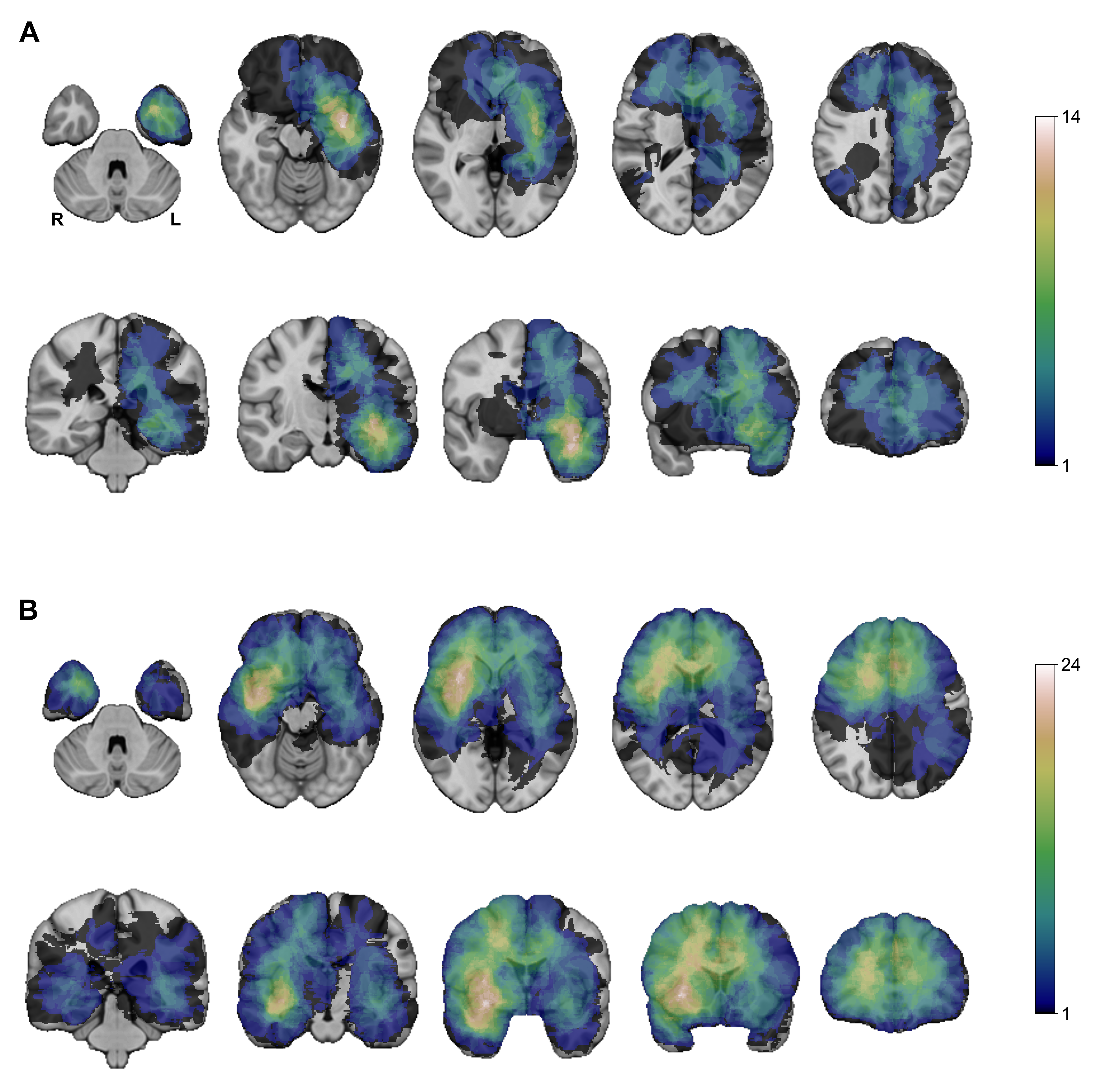

Supplement: noaf263_Supplementary_Data [file noaf263_supplementary_data.zip › SupplementaryFigure3.tiff]
